# SIRT1 inactivation switches reactive astrocytes to an antiinflammatory phenotype in CNS autoimmunity

**DOI:** 10.1172/JCI151803

**Published:** 2022-11-15

**Authors:** Weifeng Zhang, Dan Xiao, Xing Li, Yuan Zhang, Javad Rasouli, Giacomo Casella, Alexandra Boehm, Daniel Hwang, Larissa L.W. Ishikawa, Rodolfo Thome, Bogoljub Ciric, Mark T. Curtis, Abdolmohamad Rostami, Guang-Xian Zhang

**Affiliations:** 1 Department of Neurology, Thomas Jefferson University, Philadelphia, Pennsylvania, USA.; 2 Department of Biochemistry, College of Life Sciences, Shaanxi Normal University, Xi’an, Shaanxi, China.; 3 Department of Pathology, Thomas Jefferson University, Philadelphia, Pennsylvania, USA.

**Keywords:** Autoimmunity, Inflammation, Autoimmune diseases, Multiple sclerosis

## Abstract

Astrocytes are highly heterogeneous in their phenotype and function, which contributes to CNS disease, repair, and aging; however, the molecular mechanism of their functional states remains largely unknown. Here, we show that activation of sirtuin 1 (SIRT1), a protein deacetylase, played an important role in the detrimental actions of reactive astrocytes, whereas its inactivation conferred these cells with antiinflammatory functions that inhibited the production of proinflammatory mediators by myeloid cells and microglia and promoted the differentiation of oligodendrocyte progenitor cells. Mice with astrocyte-specific *Sirt1* knockout (*Sirt1^–/–^*) had suppressed progression of experimental autoimmune encephalomyelitis (EAE), an animal model of CNS inflammatory demyelinating disease. Ongoing EAE was also suppressed when *Sirt1* expression in astrocytes was diminished by a CRISPR/Cas vector, resulting in reduced demyelination, decreased numbers of T cells, and an increased rate of IL-10–producing macrophages and microglia in the CNS, whereas the peripheral immune response remained unaffected. Mechanistically, *Sirt1^–/–^* astrocytes expressed a range of nuclear factor erythroid–derived 2–like 2 (*Nfe2l2*) target genes, and *Nfe2l2* deficiency shifted the beneficial action of *Sirt1^–/–^* astrocytes to a detrimental one. These findings identify an approach for switching the functional state of reactive astrocytes that will facilitate the development of astrocyte-targeting therapies for inflammatory neurodegenerative diseases such as multiple sclerosis.

## Graphical abstract

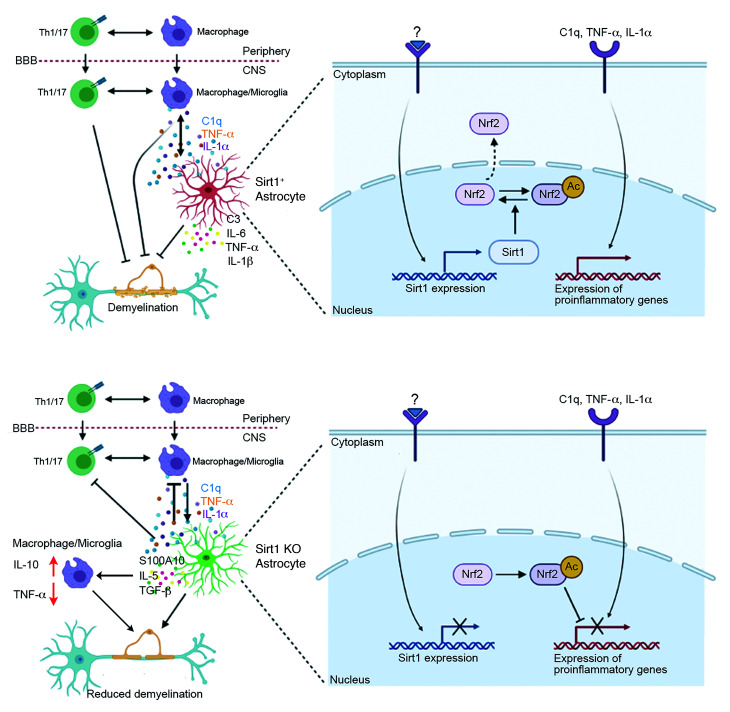

## Introduction

Astrocytes, the most abundant cell type in the mammalian CNS, have been considered both an ally and enemy in the fight against CNS inflammation and restoration of neuronal function in multiple sclerosis (MS) (1–3). Astrocytes support neural transmission, sustain the survival of neurons and other glia, and maintain the integrity of the blood-brain barrier (BBB) (1–3). Astrocyte scar formation aids, rather than prevents, CNS axon regeneration after spinal cord injury (4). Astrocytes also promote oligodendrocyte maturation and remyelination through the production of neurotrophic factors such as CNTF (5) and BDNF (6). Conversely, astrocytes are viewed as important nonprofessional antigen-presenting cells, and, during CNS inflammation, astrocytes around the MS lesion produce multiple proinflammatory mediators, including vasoactive molecules, chemoattractants, adhesion molecules, and cytokines that increase BBB permeability and promote lymphocyte recruitment, activation, and survival (1, 7). Reactive astrocytes undergo morphological, molecular, and functional remodeling in response to various pathological factors, and those toxic to neurons and oligodendrocytes have been classified as A1 astrocytes, whereas A2 astrocytes are probably neuroprotective, given their production of many neurotrophic factors (8). However, this binary classification may not well recapitulate the heterogeneity of astrocyte activation states (9). Astrocyte subsets have been more recently defined by their molecular signatures using more contemporary molecular techniques, e.g., single-cell RNA-Seq (scRNA-seq), and/or their function, which may reflect various sources of astrocyte heterogeneity (3). Understanding the mechanisms underlying astrocyte heterogeneity and plasticity may yield therapeutic approaches for switching astrocyte subsets from pathogenic to beneficial in neurological disorders such as MS.

Sirtuins (SIRTs), which are members of the class III histone/lysine deacetylase family, play critical roles in transcriptional regulation, cell cycling, replicative senescence, inflammation, and metabolism (10, 11). Among them, SIRT1 is expressed in many tissues and cell types and acts as an epigenetic regulator that modulates the activity of several transcription factors important for immune function (12). While SIRT1 may have an antiinflammatory role systemically (12, 13), likely via deacetylation of FOXP3 (14, 15), proinflammatory roles of SIRT1 have also been reported (16–18), whereby T cell– (19) or DC-specific (20) SIRT1 deletion protected mice from experimental autoimmune encephalomyelitis (EAE), an animal model of CNS inflammatory demyelinating disease. In the nervous system, SIRT1 plays a protective role in neurons (21–23) but an inhibitory role in oligodendrocyte progenitor cell (OPC) proliferation without affecting their differentiation (24, 25). The role of SIRT1 in astrocytes remains controversial, being either beneficial (22, 26, 27) or detrimental (25, 28, 29), whereas its role in astrocytes in MS and EAE remains unknown. In the present study, we have defined the role of SIRT1 in the molecular profile and function of astrocytes and explored the potential of targeting SIRT1 in these cells as a CNS-specific therapy for MS.

## Results

### Inactivation of SIRT1 promotes an antiinflammatory phenotype in astrocytes in vitro.

**Figure 1:**
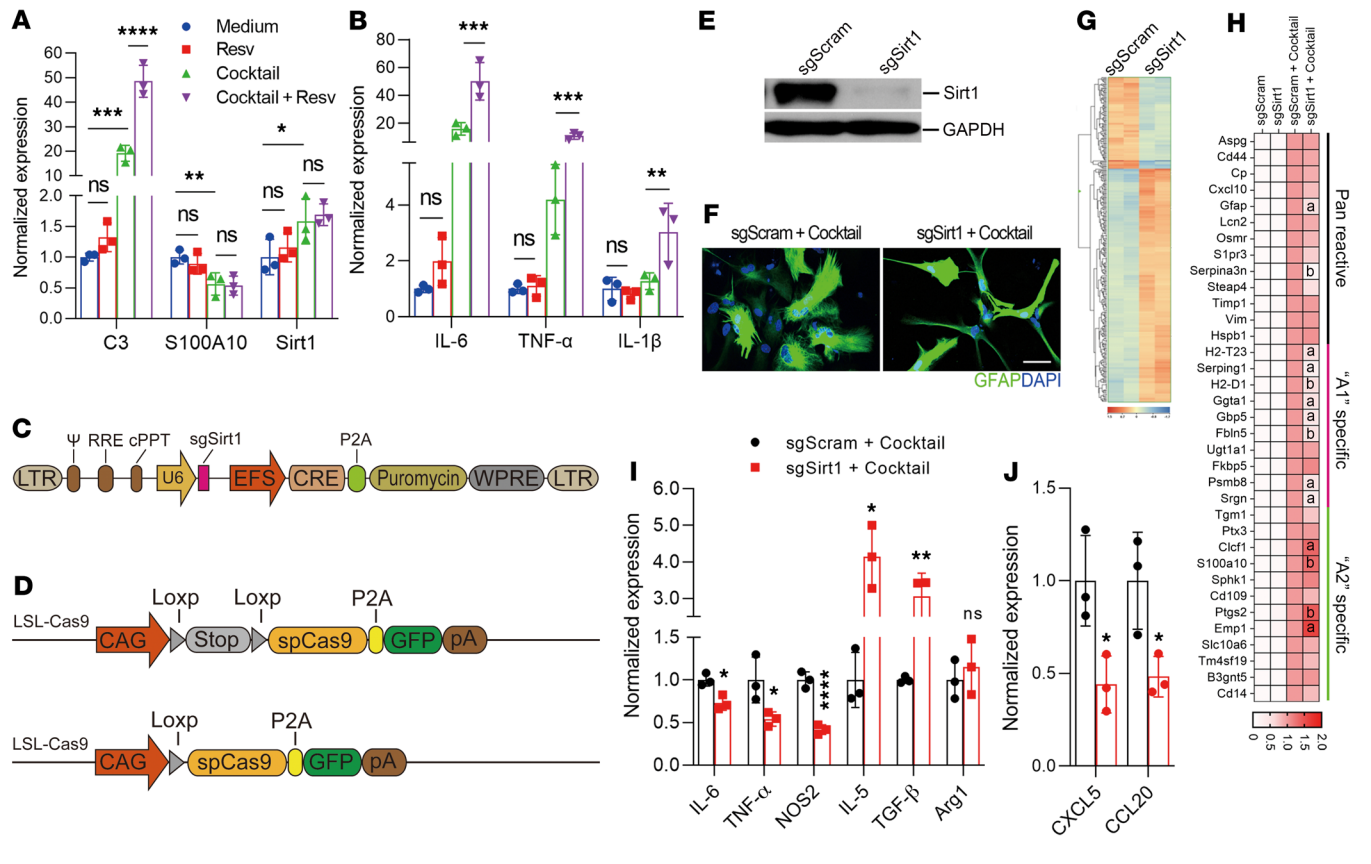
Inactivation of SIRT1 promotes antiinflammatory astrocytes. (**A** and **B**) Primary astrocytes were isolated from naive newborn C57BL/6 mice at P2, treated with resveratrol (Resv) or DMSO for 24 hours, and then stimulated with cocktail (C1q, TNF, IL-1α) for another 24 hours. Expression of representative A1/A2 astrocyte markers (**A**) and proinflammatory cytokines (**B**) was determined by RT-PCR. *n* = 3 per group; 1-way ANOVA. (**C**–**G**) Primary astrocytes isolated from LSL-Cas9 mice (**D**) were infected with lentivirus carrying *Cre* with sgSirt1 or sgScram (**C**). Forty-eight hours after infection, cells were selected with puromycin for 3 days, and knockout efficiency was analyzed by Western blotting (**E**). sgSirt1- and sgScram-treated astrocytes were stimulated with the cocktail for 24 hours and assayed by immunostaining for their morphology (**F**), by microarray for the RNA expression profiles (**G**), and by RT-PCR to measure the expression of neurotoxic (“A1”), neuroprotective (“A2”), and pan-reactive astrocyte markers (8) (**H**), pro- or antiinflammatory molecules (**I**), and chemokines (**J**). *n* = 3 per group; unpaired, 2-tailed *t* test. All results are expressed as the mean ± SD. *P < 0.05 (a), **P < 0.01 (b), ***P < 0.001 (c), and ****P < 0.0001 (d). Data from 1 representative experiment of 3 are shown.

To study the impact of SIRT1 on the phenotype and function of astrocytes, we first activated SIRT1 in primary astrocytes with resveratrol. Primary mouse astrocytes were prepared from C57BL/6 mice at P2–P3 (30), with GFAP^+^ cell homogeneity above 98% as determined by flow cytometry (Supplemental Figure 2; supplemental material available online with this article; https://doi.org/10.1172/JCI151803DS1). Activation with resveratrol alone did not affect gene expression in astrocytes; however, it significantly enhanced a cocktail-induced (C1q, IL-1α, and TNF) expression of complement component 3 (C3), a representative marker for neurotoxic astrocytes (8) (Figure 1A), together with the proinflammatory cytokines IL-6, TNF, and IL-1β (Figure 1B). In contrast, S100A10, a probable marker for neuroprotective astrocytes (8), remained unchanged (Figure 1A).

Next, we tested whether the absence of SIRT1 affects the gene expression and function of astrocytes. Four sgRNAs targeting exon4 of mouse *Sirt1* were synthesized, their knockout efficiencies tested in the N2A-Cas9 cell line, and the most efficient sgRNA subcloned into a lentivirus carrying *Cre* and puromycin genes driven by the EFS promoter (Figure 1C). We isolated primary astrocytes from LSL-Cas9–transgenic mice carrying a Cas9-P2A-GFP cassette driven by the CAG promoter, which was blocked by a floxed stop signal (Figure 1D). In this system, infection with the lentivirus carrying the *Cre* gene can induce the expression of CAS9 in astrocytes, which, in combination with sgRNA expressed from lentivirus, cleaves the targeted sequence and leads to its knockout (31). *Sirt1* was knocked out in astrocytes with high efficiency, as shown by Western blotting (Figure 1E). While lenti-sgScram–infected astrocytes treated with the cocktail developed an activated morphology, e.g., cellular hypertrophy, and decreased fine processes (Figure 1F) with increased expression of glial fibrillary acidic protein (GFAP) (32) (Figure 1H), astrocytes with *Sirt1* knockout had a less activated morphology (Figure 1F). The RNA expression profile of astrocytes was substantially changed by *Sirt1* knockout as detected by microarray analysis (Figure 1G). When further validated by real-time PCR (RT-PCR), we observed reduced expression of cocktail-induced neurotoxic astrocyte markers and enhanced neuroprotective astrocyte markers in *Sirt1^–/–^* astrocytes; however, there were no differences between *Sirt1*-sufficient and *Sirt1*-deficient astrocytes in the nonstimulated condition (Figure 1H). *Sirt1* knockout also reduced the expression of the proinflammatory molecules NOS2, IL-6, and TNF and enhanced the expression of immunomodulatory molecules Arg1, IL-5, and TGF-β by cocktail-stimulated astrocytes (Figure 1I). Further, *Sirt1* knockout in astrocytes reduced their expression of the chemokines CXCL5 and CCL20 (Figure 1J), which are important for CNS infiltration and proinflammatory functions of immune cells (33). These results suggest that *Sirt1* expression contributed to the development of the neurotoxic/inflammatory state of astrocytes and that its inactivation promoted an antiinflammatory state.

### Reduced EAE progression and CNS inflammation in astrocyte-specific Sirt1^–/–^ mice.

**Figure 2:**
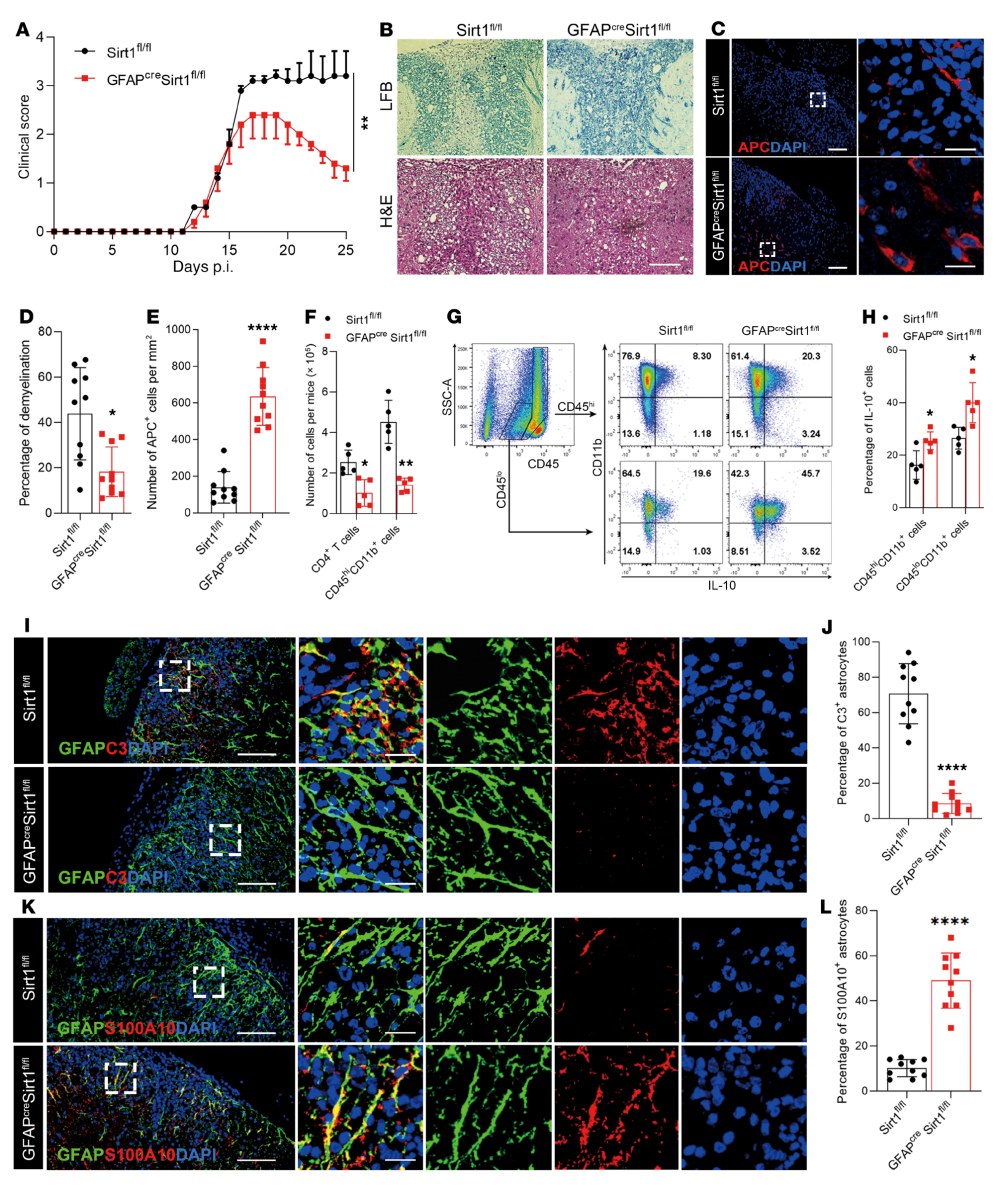
Astrocyte-specific knockout of *Sirt1* suppresses EAE progression. EAE was induced in GFAP^Cre^ *Sirt1^fl/fl^* mice and *Sirt1^fl/fl^* control mice by MOG_35–55_ peptide in CFA and pertussis toxin. (**A**) Clinical signs were scored in a blinded manner by 2 researchers following a 0–5 scale. Mice were sacrificed on day 25 p.i. *n* = 5 mice per group. Results are expressed as the mean ± SEM. (**B**) Representative LFB- and H&E-stained images of spinal cord from EAE mice. Scale bar: 50 μm. (**C**) Immunofluorescence staining of spinal cord from EAE mice with anti-APC antibody (CC1), a marker of newly formed oligodendrocytes. Scale bars: 100 μm (left panel) and 20 μm (enlarged insets, right panel). (**D** and **E**) Statistical analysis of LFB (**D**) and APC (**E**) staining results. *n* = 5 mice per group. (**F**) Total number of CD4^+^ T cells and myeloid cells (CD45^hi^CD11b^+^) from the CNS of GFAP^Cre^ *Sirt1^fl/fl^* and *Sirt1^fl/fl^* mice were analyzed by flow cytometry. (**G**) Expression of IL-10 in CD45^hi^ (myeloid) and CD45^lo^ (microglia) cells from GFAP^Cre^ *Sirt1^fl/fl^* or *Sirt1^fl/fl^* EAE mice was analyzed by flow cytometry. (**H**) Statistical analysis of the data in **G**. *n* = 5 mice per group. (**I**–**L**) Spinal cords were stained for GFAP and C3, a representative marker of neurotoxic astrocytes (**I**), as well as GFAP and S100A10, a representative marker of antiinflammatory astrocytes (**K**). Scale bars: 100 μm (left panels in **I** and **K**) and 20 μm (enlarged insets in the right panels in **I** and **K**). The percentages of C3^+^ (**J**) and S100A10^+^ (**L**) astrocytes were quantified. *n* = 5 mice per group. Data in **D**–**F**, **H**, **J**, and **L** are expressed as the mean ± SD. *P < 0.05, **P < 0.01, and ****P < 0.0001, by 2-way, repeated-measures ANOVA (**A**) and unpaired, 2-tailed *t* test (**D**–**F**, **H**, **J**, and **L**). Data from 1 representative experiment of 3 are shown.

To evaluate the role of SIRT1 in astrocytes in EAE, we induced EAE in mice lacking SIRT1 in GFAP^+^ cells (GFAP^Cre^ *Sirt1^fl/fl^* mice). These mice initially developed EAE similar to that in the control *Sirt1^fl/fl^* mice but then gradually recovered from the disease (Figure 2A). Histopathological analyses showed reduced immune cell infiltration and demyelination (Figure 2, B and D), with increased numbers of adenomatous polyposis coli–positive (APC^+^) cells (newly matured oligodendrocytes) in the CNS lesions of GFAP^Cre^ *Sirt1^fl/fl^* mice compared with the control mice (Figure 2, C and E). Mononuclear cells (MNCs) isolated from the CNS were analyzed by flow cytometry. The total number of CD4^+^ T cells and myeloid cells was largely reduced in GFAP^Cre^ *Sirt1^fl/fl^* mice (Figure 2F), with increased percentages of IL-10^+^ myeloid cells (CD11b^+^CD45^hi^) and microglia (CD11b^+^CD45^lo^) (Figure 2, G and H). The frequencies of various CD4^+^ T cell subtypes in the CNS were not different between these 2 mouse lines (Supplemental Figure 3, A and B). We observed reduced numbers of C3^+^ astrocytes (Figure 2, I and J) and increased numbers of S100A10^+^ astrocytes (Figure 2, K and L) in the CNS lesions of GFAP^Cre^ *Sirt1^fl/fl^* mice compared with *Sirt1^fl/fl^* mice. In contrast, no difference between these 2 groups was observed in the total numbers or subsets of monocytes and CD4^+^ T cells in their spleens (Supplemental Figure 4, A–D) and draining lymph nodes (data not shown). These results show that SIRT1 in astrocytes plays an important role in EAE progression, possibly by promoting a neurotoxic/inflammatory astrocyte phenotype; furthermore, its inactivation enhances antiinflammatory astrocytes and suppresses CNS autoimmunity without affecting the peripheral immune response.

### Sirt1^–/–^ astrocytes induced immunomodulatory microglia and macrophages and enhanced OPC differentiation.

**Figure 3:**
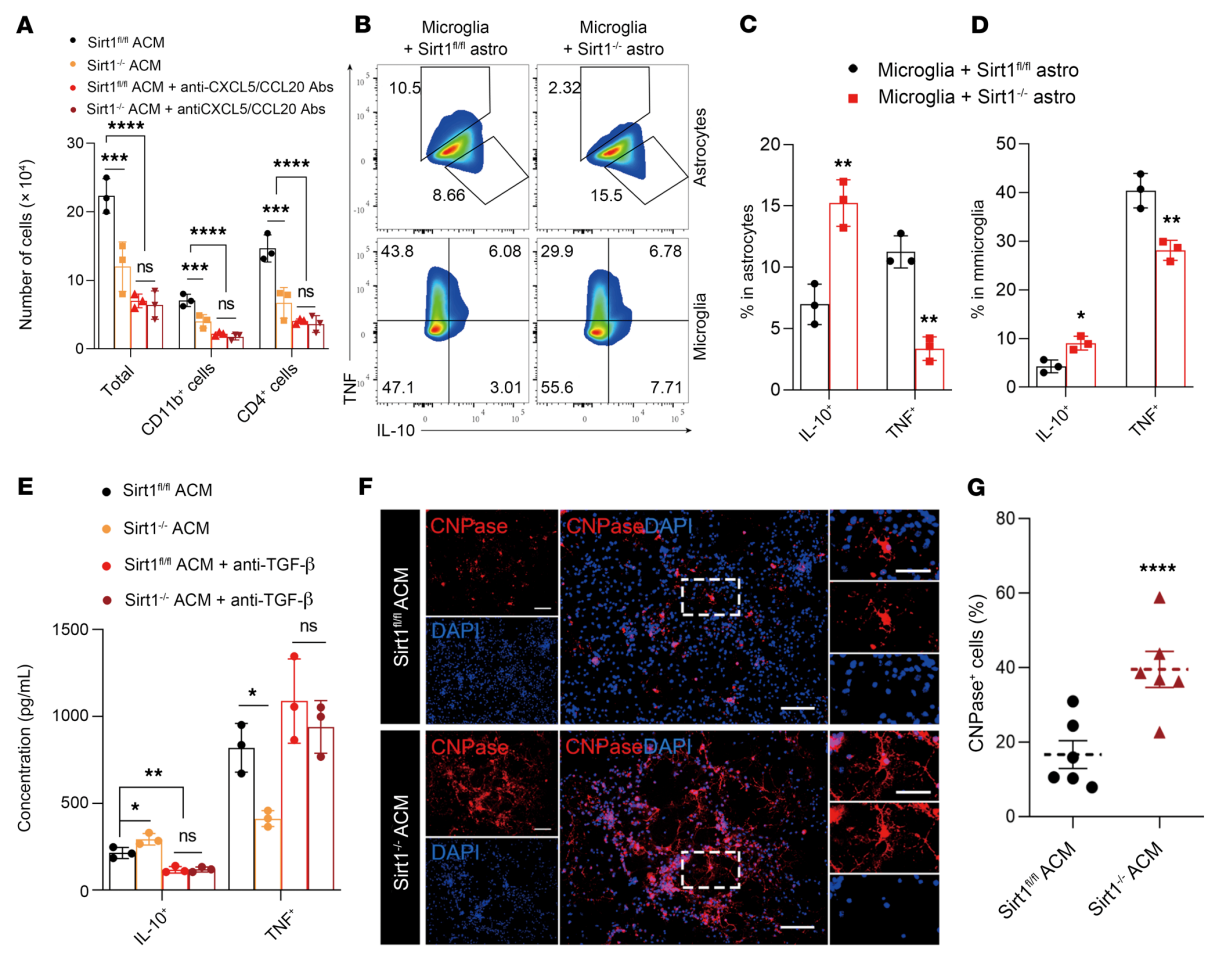
Astrocyte-specific *Sirt1^–/–^* reduces migration and inflammation of immune cells and enhances OPC differentiation. Astrocytes isolated from the brains of newborn GFAP^Cre^ *Sirt1^fl/fl^* or *Sirt1^fl/fl^* mice were stimulated with cocktail for 24 hours, washed, and cultured in fresh medium. These astrocytes were then cultured for an additional 24 hours to collect supernatant (ACM). (**A**) Splenocytes of WT EAE mice were harvested on day 12 p.i. and cultured with ACMs from *Sirt1^–/–^* or WT astrocytes using a Transwell cell culture insert. Cells in the bottom chamber were harvested 2 hours later, and the migration of CD4^+^ T cells and CD11b^+^ cells was analyzed by flow cytometry. *n* = 3 samples per group. (**B**) Microglia were isolated from the brains of newborn WT mice, preactivated with LPS for 18 hours, and cocultured with cocktail-stimulated *Sirt1^–/–^* or WT astrocytes for 24 hours. TNF and IL-10 production by astrocytes and microglia was analyzed by flow cytometry. (**C** and **D**) Statistical analysis of the data in **B**. *n* = 3 samples per group. (**E**) LPS-stimulated microglia were incubated in *Sirt1^–/–^* or control ACMs with or without anti–TGF-β–neutralizing antibody for 24 hours and then washed and cultured in fresh medium for an additional 24 hours, after which the concentrations of TNF and IL-10 in the culture supernatants were measured by ELISA. *n* = 3 samples per group. (**F** and **G**) OPCs were generated from brains of newborn WT mice, cultured in differentiation medium that was supplemented with ACMs of cocktail-stimulated *Sirt1^–/–^* or WT astrocytes for 8 days, and stained for myelin basic protein (MBP). *n* = 3 samples per group. Scale bars: 50 μm and 25 μm (enlarged insets). All results are expressed as the mean ± SD. *P < 0.05, **P < 0.01, ***P < 0.001, and ****P < 0.0001, by unpaired, 1-way ANOVA (**A** and **E**) and 2-tailed *t* test (**C**, **D**, and **G**). Data from 1 representative experiment of 3 are shown.

We then determined the direct impact of astrocyte *Sirt1* on microglia and macrophages and oligodendrocytes in vitro. Given the reduced number of immune cells in the CNS of astrocyte-specific *Sirt1^–/–^* EAE mice (Figure 2F) and the reduced secretion of CXCL5 and CCL20 in *Sirt1^–/–^* astrocytes (Figure 1J), we tested the cause-effect relationship of these 2 phenomena in vitro. We tested the migration of splenocytes from mice with EAE toward astrocyte-conditioned medium (ACM) of cocktail-stimulated *Sirt1^–/–^* and WT astrocytes in a Transwell system. Fewer total MNCs, CD11b^+^ myeloid cells, and CD4^+^ T cells migrated toward the ACMs of *Sirt1^–/–^* astrocytes than toward the ACMs of WT astrocytes, and this migration was reduced by neutralizing anti-CXCL5 and anti-CCL20 antibodies (Figure 3A).

*Sirt1^–/–^* mice had increased IL-10 secretion by microglia and myeloid cells in the CNS during EAE (as shown in Figure 2, G and H). To test whether this was a direct consequence of Sirt1 knockout in astrocytes, we preactivated primary microglia from WT mice with LPS and stimulated *Sirt1^–/–^* or WT astrocytes with the cocktail (C1q, IL-1α, TNF). We then cocultured these cells and analyzed their expression of IL-10 and TNF by flow cytometry. *Sirt1* knockout reduced the percentage of TNF^+^ astrocytes and increased the percentage of IL-10^+^ astrocytes (Figure 3, B and C). Similarly, we observed a reduced percentage of TNF^+^ microglia and an increased percentage of IL-10^+^ microglia when they were cocultured with *Sirt1^–/–^* astrocytes (Figure 3, B and D). Given the enhanced expression of TGF-β by *Sirt1^–/–^* astrocytes (Figure 1I), which can influence the activation (34) and cytokine production of microglia (Supplemental Figure 5), we speculated that *Sirt1^–/–^* astrocytes could induce immunomodulatory microglia through increased TGF-β secretion. To test this, we incubated LPS-activated microglia with supernatants from cocktail-stimulated *Sirt1^–/–^* or WT astrocytes, with or without adding TGF-β–neutralizing antibody to the cultures. Whereas supernatants from *Sirt1^–/–^* astrocytes reduced TNF and increased IL-10 secretion by microglia, we found that these effects were blocked by TGF-β–neutralizing antibodies (Figure 3E). Together, these results indicate that *Sirt1^–/–^* astrocytes directly affected the function of activated microglia, and immunomodulatory mediators such as TGF-β produced by these astrocytes played an important role in this effect.

We also analyzed the direct effect of ACMs from *Sirt1^–/–^* versus WT astrocytes on the differentiation of OPCs. While OPCs cultured with ACMs from cocktail-stimulated WT astrocytes exhibited a low level of differentiation (<20% CNPase^+^ mature oligodendrocytes) this proportion was significantly increased (>40%) in those cultured with ACMs of *Sirt1^–/–^* astrocytes (Figure 3, F and G). Taken together, these results show that *Sirt1^–/–^* astrocytes reduced immune cell migration and proinflammatory microglia polarization while enhancing OPC differentiation.

### Sirt1^–/–^ astrocytes acquired antiinflammatory function through NRF2 activation.

**Figure 4:**
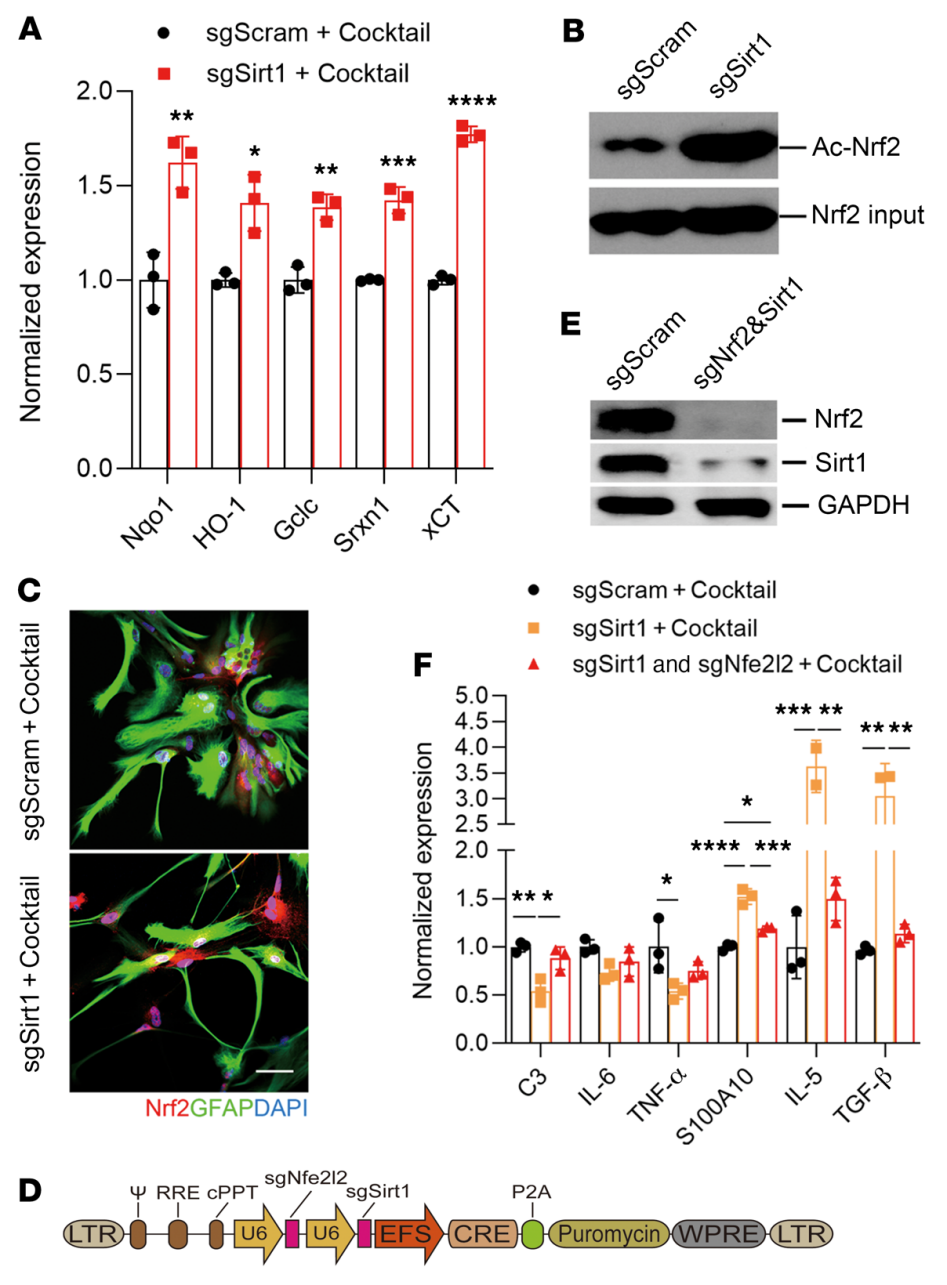
SIRT1 enhances A1 astrocytes by inhibiting the activity of NRF2. mRNA was extracted from sgSirt1- or sgScram-treated astrocytes after stimulation with the cocktail containing C1q, IL-1α, and TNF for 24 hours and used for RT-PCR analysis. (**A**) Expression of NRF2 target genes as determined by RT-PCR. *n* = 3 per group. (**B**) IP analysis of NRF2 acetylation in sgSirt1- and sgScram-treated astrocytes. (**C**) Immunostaining for NRF2 in astrocytes. Scale bar: 50 μm. (**D**–**F**) Primary astrocytes were transduced with sgSirt1, sgSirt1 and sgNfe2l2, or sgScram vectors and then stimulated with the cocktail for 24 hours. (**D**) Structure of the lentivirus carrying sgSirt1 and sgNfe2l2. (**E**) Western blot verification of *Sirt1* and *Nfe2l2* knockout. (**F**) The expression of certain reactive astrocyte markers and cytokines was determined by RT-PCR. *n* = 3 per group. All results are expressed as the mean ± SD. *P < 0.05 (a), **P < 0.01 (b), ***P < 0.001 (c), and ****P < 0.0001 (d), by unpaired, 2-tailed *t* test (**A**) and 1-way ANOVA (**F**). Data from 1 representative experiment of 3 are shown.

To define the mechanism underlying the role of *Sirt1* in astrocytes, we further analyzed the RNA expression profiles of *Sirt1^–/–^* versus WT astrocytes. Among the altered genes shown in Figure 1G, *Sirt1^–/–^* astrocytes expressed increased levels of NQO1, xCT (*SLC7A11*), Srxn1, Gclc, and HO-1 (*HMOX1*), all of which are targets of the nuclear factor erythroid 2–related factor 2 (NRF2) transcription factor (35). Their expression was further confirmed by RT-PCR (Figure 4A); however, expression of the *Nfe2l2* gene remained unchanged (data not shown). NRF2 protein, encoded by the *Nfe2l2* gene, is a transcription factor that has important antiinflammatory and antioxidant functions (36). Given that acetylation of NRF2 is important for its function (37), we tested the effect of SIRT1 on NFR2 acetylation and found that *Sirt1^–/–^* astrocytes had greatly enhanced NRF2 acetylation (Figure 4B) localized in the nucleus (Figure 4C). To test the role of NRF2 in SIRT1-mediated astrocyte function, we simultaneously knocked out *Nfe2l2* and *Sirt1* out in primary WT astrocytes using the CRISPR/Cas technique (Figure 4D), and confirmed the knockout efficacy by Western blotting (Figure 4E). Knockout of *Nfe2l2* abrogated the antiinflammatory phenotype of *Sirt1^–/–^* astrocytes, as shown by increased expression of C3 and decreased expression of S100A10, IL-5, and TGF-β (Figure 4F). Thus, the NRF2 signaling pathway plays an important role in the antiinflammatory transcriptional programs of *Sirt1^–/–^* astrocytes.

### Adeno-associated virus-CRISPR–mediated knockout of Sirt1 in astrocytes promoted recovery in ongoing EAE.

**Figure 5:**
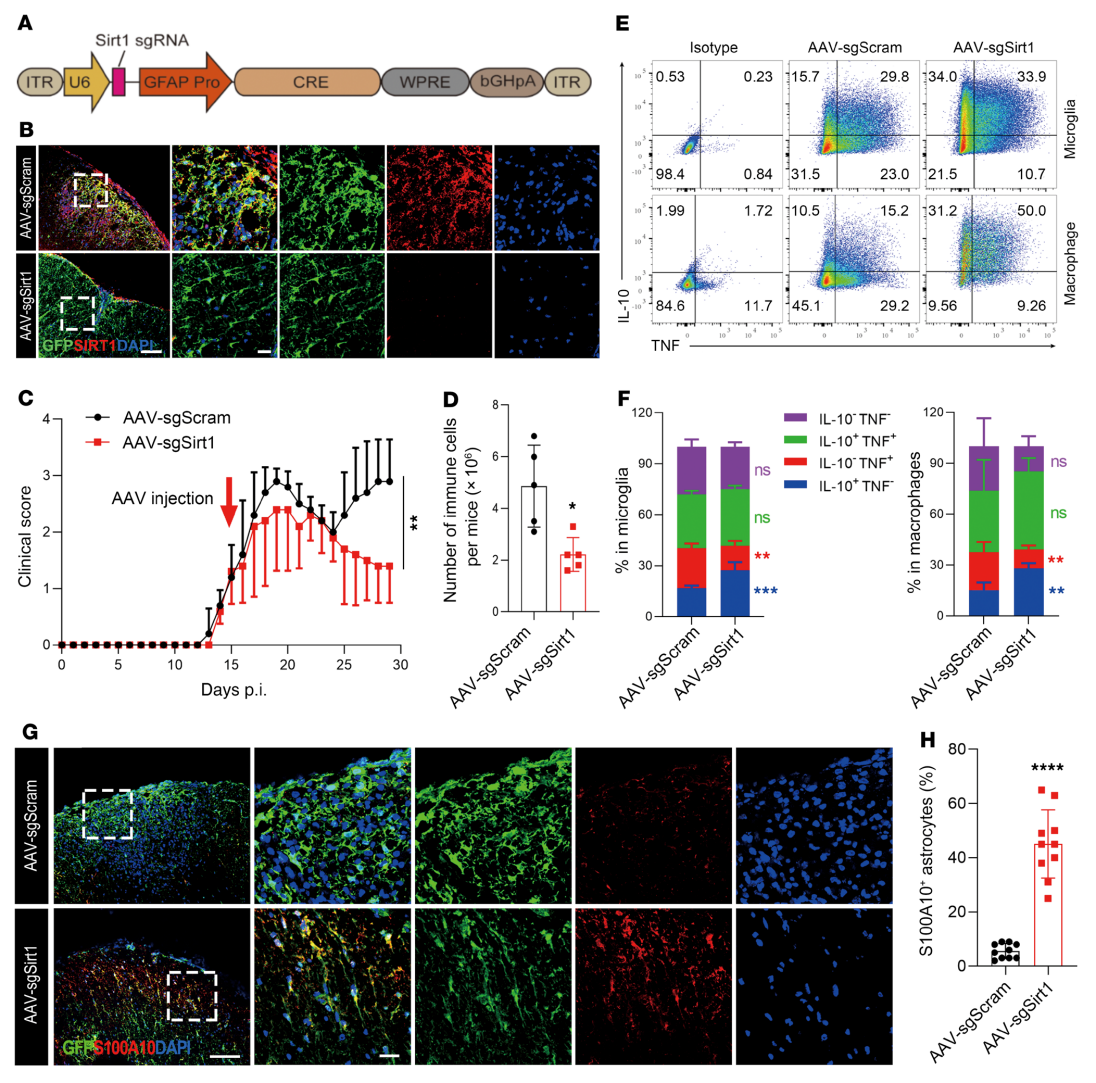
CRISPR/Cas9-mediated astrocyte-specific *Sirt1* knockout effectively alleviates ongoing EAE. (**A**) Structure of the AAV plasmid for *Sirt1* knockout in vivo. (**B**–**H**) EAE was induced in LSL-Cas9 mice (on a C57BL/6 background) by MOG_35–55_ peptide in CFA and pertussis toxin. AAV carrying *Sirt1* sgRNA or scrambled sgRNA was injected through the tail vein on day 15 p.i. Mice were sacrificed on day 30 p.i., and brains, spinal cords, and spleens were harvested. (**B**) Knockout efficiency of *Sirt1* in astrocytes was determined by immunostaining of lumbar spinal cord. Scale bars: 100 μm and 20 μm (enlarged insets). (**C**) EAE score of AAV-sgSirt1– and AAV-sgScram–injected mice. *n* = 5 mice for the AAV-sgSirt1 group; *n* = 8 mice for the AAV-sgScram group. (**D**) Statistical analysis of the number of MNCs in the CNS of mice with EAE. (**E**) Flow cytometric analysis of the percentages of different phenotypes of microglia (CD45^lo^CD11^+^) and macrophages (CD45^hi^CD11^+^) in the CNS of mice with EAE. (**F**) Statistical analysis of the data in **E**. (**G**) Spinal cords from AAV-sgSirt1– and AAV-sgScram–injected mice with EAE were costained for GFP (to detect AAV-infected astrocytes) and S100A10. Scale bars: 100 μm and 20 μm (enlarged insets). (**H**) Statistical analysis of the percentages of S100A10^+^ astrocytes. *n* = 5 mice per group (**D**–**H**). All results are expressed as the mean ± SD. *P < 0.05, **P < 0.01, ***P < 0.001, and ****P < 0.0001, by 2-way, repeated-measures ANOVA (**C**) and unpaired, 2-tailed *t* test (**D**, **F**, and **H**). Data from 1 representative experiment of 2 are shown.

**Figure 6:**
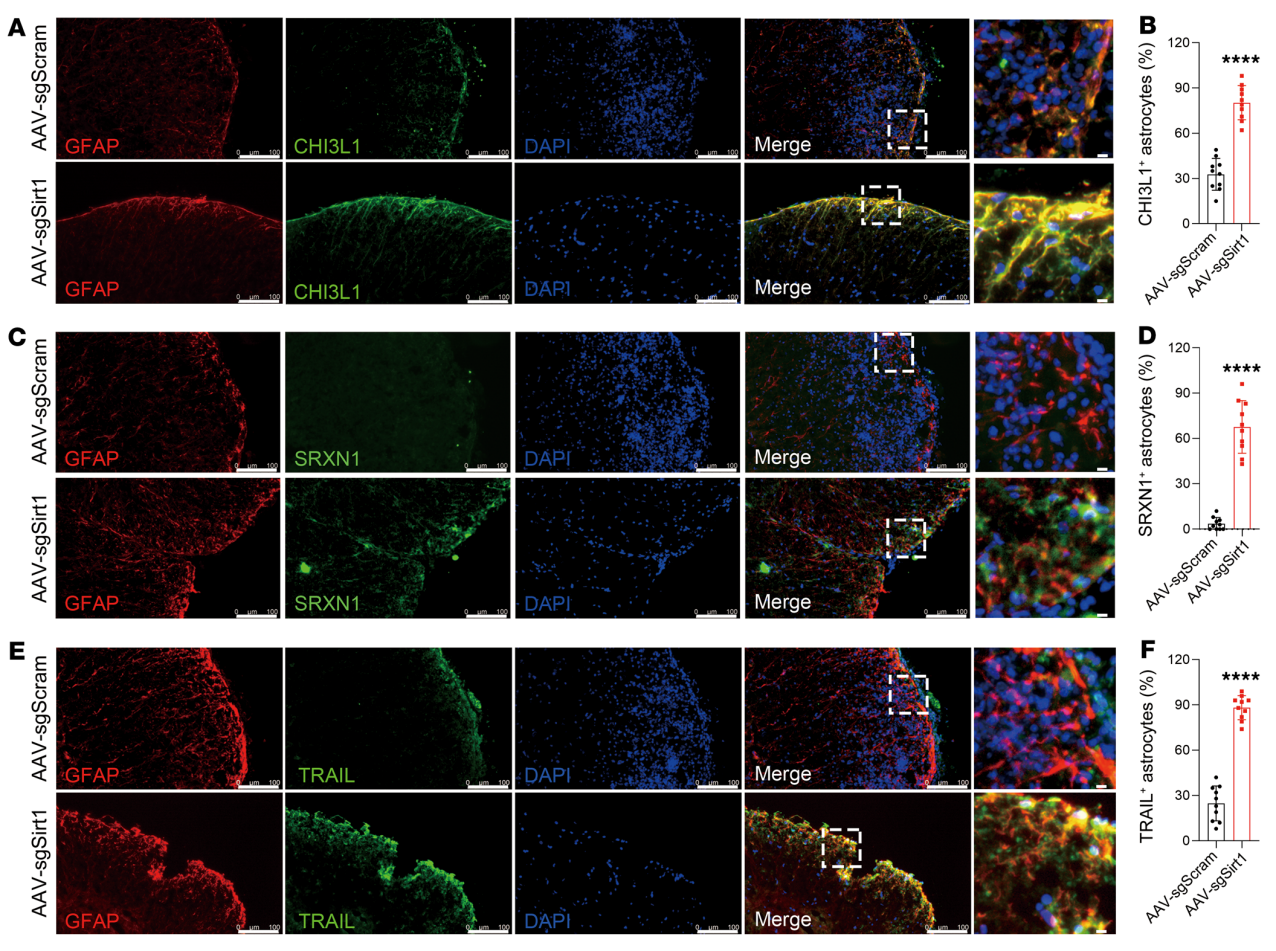
Immunostaining analysis of astrocytes in astrocyte-specific *Sirt1*-knockout and WT EAE mice. Spinal cords from AAV-sgSirt1– and AAV-sgScram–injected mice with EAE were costained for GFAP and CHI3L1 (**A**), SRXN1 (**C**), and TRAIL (**E**). Scale bars: 100 μm and 100 μm (enlarged insets). Analysis of costaining results for CHI3L1 (**B**), SRXN1 (**D**), and TRAIL (**F**). *n* = 5 mice per group, unpaired, 2-tailed *t* test. All results are expressed as the mean ± SD. ****P < 0.0001, by unpaired, 2-tailed *t* test. Data from 1 representative experiment of 2 are shown.

To test whether astrocyte-specific *Sirt1* knockout can alleviate ongoing EAE, we generated an adeno-associated virus (AAV) transfer plasmid carrying a *Cre* gene driven by a GFAP promoter and *Sirt1*-targeting sgRNA (Figure 5A). PHP.eB, a newly developed AAV serotype that efficiently transduces the CNS via systemic delivery in adult animals (38), was used to achieve astrocyte-specific knockout of *Sirt1* in the entire CNS (39). EAE was induced in LSL-Cas9 transgenic mice, and AAV was i.v. injected on day 15 post immunization (p.i.), when disease was still worsening. We confirmed the efficacy of *Sirt1* knockout in astrocytes (GFP^+^) by immunostaining (Figure 5B). Although both groups experienced a temporary reduction in disease severity, likely due to a nonspecific effect of vector injection (40), the clinical score had increased in mice after 10 days of control AAV injection. In contrast, disease severity remained suppressed in mice with astrocyte-specific knockout of *Sirt1* (Figure 5C), with greatly reduced infiltration of immune cells into the CNS (Figure 5D). AAV-sgSirt1 treatment increased the numbers of IL-10^+^ and reduced the numbers of TNF^+^ microglia and macrophages (Figure 5, E and F), whereas the percentages of CD4^+^ T cell subtypes remained similar (Supplemental Figure 6). Furthermore, we found significantly increased percentages of GFAP^+^ astrocytes colocalized with S100A10 in astrocytes from mice in the AAV-sgSirt1–treated group (Figure 5, G and H). The AAV-sgSirt1–treated group also had upregulated expression of antiinflammatory molecules in astrocytes, including chitinase 3 like 1 (CHI3L1) (41–43), sulfiredoxin-1 (SRXN1) (44), and TNF-related apoptosis-inducing ligand (TRAIL) (45) (Figure 6). In contrast, there was no difference in peripheral immune responses between these 2 groups (Supplemental Figure 7). These results demonstrate that CRISPR-mediated *Sirt1* knockout in astrocytes after disease onset resulted in an antiinflammatory profile that promoted recovery in ongoing EAE and thus has the potential for clinical application.

### SIRT1 is highly expressed in C3^+^ astrocytes of patients with MS.

**Figure 7:**
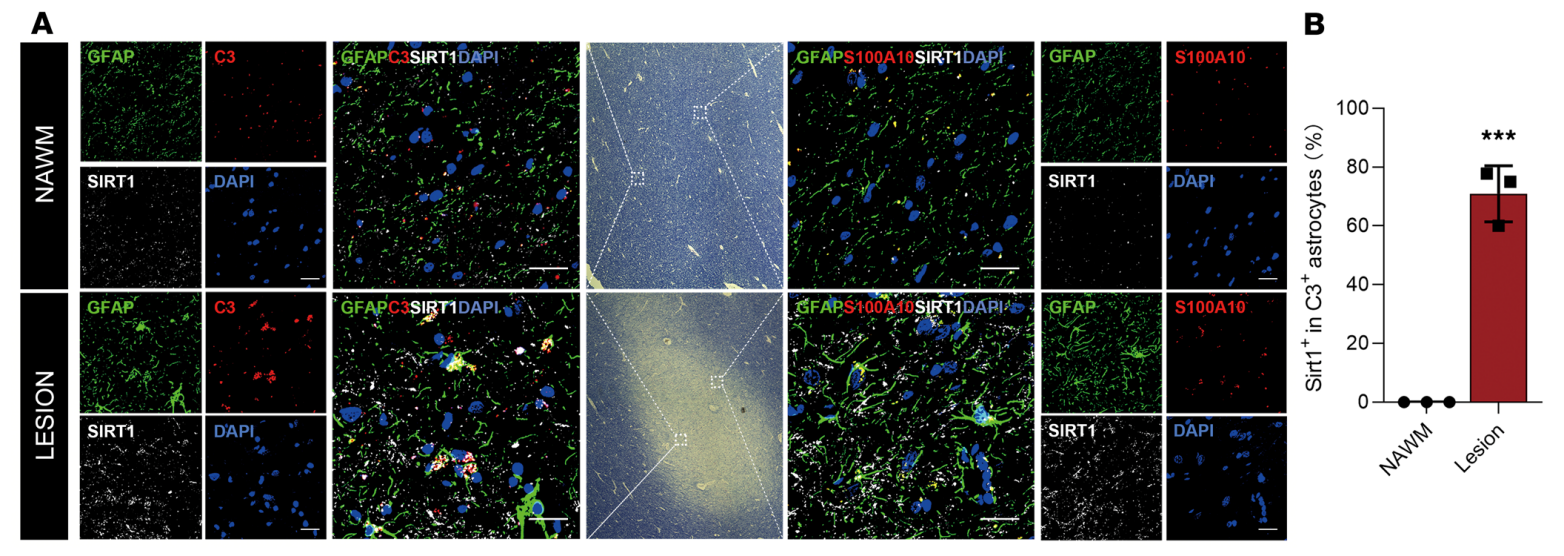
SIRT1 is highly expressed in A1 astrocytes in the lesions of patients with MS. (**A**) Brain tissues from patients with MS were coimmunostained for GFAP, SIRT1, and DAPI with C3 or S100A10, and NAWM served as a control. The left and right panels are the higher magnifications of the insets shown in the middle panel. Scale bars: 100 μm (merged images) and 100 µm (individual images of channels). (**B**) Statistical analysis. For quantification, lesion tissues from the brains of 3 patients with MS were examined. Nine sections (3 per lesion) were randomly selected and quantitated. The number of stained cells per section was counted under ×40 magnification. Data are expressed as the mean ± SD. ***P < 0.001, by unpaired, 2-tailed *t* test.

C3^+^ astrocytes have been found in the demyelinating plaques of patients with MS, which may inhibit OPC proliferation and differentiation and induce oligodendrocytes (8). Here, we tested the relationship of SIRT1 expression and C3/S100A10 in astrocytes of MS lesions. Brain slices from patients with MS were costained for GFAP, SIRT1, and either C3 or S100A10; normal-appearing white matter (NAWM) distant from the lesion served as a control. A large number of C3^+^SIRT1^+^GFAP^+^ cells were present in MS lesions (Figure 7A, lower panel), with nearly 71% of C3^+^ astrocytes costained for SIRT1 (Figure 7B). However, only a few S100A10^+^SIRT1^+^GFAP^+^ astrocytes were found in the lesions (Figure 7A, lower panel), and only a few astrocytes were SIRT1^+^, C3^+^, or S100A10^+^ in the NAWM area (Figure 7A, upper panel). These findings demonstrate that *Sirt1* expression is closely associated with the proinflammatory/neurotoxic astrocytes of MS lesions.

## Discussion

In the present study, we found, as summarized in Supplemental Figure 8, that reactive astrocytes expressed a high level of SIRT1, exhibited proinflammatory/neurotoxic properties, and induced CNS demyelination. Inactivation of SIRT1 converted astrocytes into a glioprotective/antiinflammatory phenotype in an NRF2-dependent manner. Genetic deletion of SIRT1 in astrocytes effectively inhibited EAE progression, and ongoing EAE was also suppressed by the astrocyte-specific *Sirt1*-knockout CRISPR/Cas vector, without affecting peripheral immune responses. These findings define an approach for inducing glioprotective/antiinflammatory astrocytes in vitro and in vivo, and provide a proof of concept for CNS-specific therapies for inflammatory neurodegenerative diseases such as MS.

It was initially thought that SIRT1 plays an antiinflammatory role (12, 13), likely via deacetylation of FoxP3, the signature transcription factor of Tregs (14, 15); however, important proinflammatory actions of SIRT1 have been recently defined. For example, inhibition of SIRT1 expression in both mouse and human T cells resulted in increased numbers of FoxP3^+^ Tregs (16–18). SIRT1 promotes autoimmunity by deacetylating RORγt, the signature transcription factor of Th17 cells, and T cell–specific *Sirt1* deletion or pharmacological inhibition of SIRT1 protects mice from EAE (19). *Sirt1^–/–^* DCs inhibit Th17 differentiation, and thereby attenuate the development of EAE (20). In the CNS, SIRT1 induces neural progenitor cell differentiation into more astrocytes but fewer neurons (25); nevertheless, SIRT1 plays a protective role in neurons (21–23). In contrast, a detrimental role of SIRT1 in the OPC/oligodendrocyte lineage has been identified. Neural stem cell–specific (NSC-specific) knockout of *Sirt1* promotes differentiation of these cells to OPCs, which can normally mature into myelinating oligodendrocytes, and mice with NSC-specific knockout of *Sirt1* showed delayed EAE onset and enhanced remyelination (24). Similar results were obtained using a SIRT1 inhibitor (25). In a neonatal brain injury model, SIRT1 inhibition promoted OPC differentiation and neuroregeneration (46). Both beneficial and detrimental roles of SIRT1 expression have been described in astrocytes: For the former, overexpression of SIRT1 attenuates astrocyte activation in vitro and improves neurobehavioral function after brain injury (22, 26). SIRT1 expression in astrocytes may have a neuroprotective effect through its antioxidative and antiinflammatory functions (27). For the latter, astrocytes with decreased expression of PPARγ and SIRT1 protect neurons from Aβ1-42 peptide–induced neurotoxicity (28). Increased numbers of oligodendrocytes have also been observed in EAE lesions after treatment with resveratrol (25, 29), a SIRT1 activator with a large range of effects, including modulation of signaling via the aryl hydrocarbon receptor (AHR) (47), NF-κB, and other molecular pathways as well (48). Our study provides evidence for a detrimental role of SIRT1 in astrocytes, given that SIRT1 inactivation enabled astrocytes to inhibit CNS inflammation and promoted OPC differentiation, thus protecting the CNS from inflammation-induced myelin damage and enhancing disease recovery.

Our findings suggest that SIRT1 regulates the functional status of reactive astrocytes, at least in part by inhibiting the expression of signaling molecules (e.g., NQO-1, xCT, Srxn1, Gclc, HO-1) that are downstream of NRF2 (35). Indeed, SIRT1 has diverse functions through its deacetylation of multiple targets, including FOXO, Ku70, p53, NF-κB, PGC-1α, RORγ, NRF2, and PPARγ (49). Among them, the transcription factor NRF2 controls cellular responses that limit oxidative stress and inflammation (50). Whole-body *Nfe2l2*-deficient mice develop severe EAE (51, 52), and *Nfe2l2*-deficient DCs induce increased proportions of activated Th1 and Th17 cells and fewer Tregs (53). Further, NRF2 activity in astrocytes could be inhibited by the proinflammatory cytokines IL-1β and TNF, and astrocyte-specific knockdown of *Nfe2l2* significantly enhances EAE severity (54). We found that *Sirt1^–/–^* astrocytes had greatly enhanced NRF2 acetylation and increased NRF2 localization in the nucleus. *Sirt1^–/–^* astrocytes had decreased expression of C3 and increased expression of S100A10, IL-5, and TGF-β compared with *Sirt1*-sufficient control astrocytes, and this profile was reversed by *Nfe2l2* knockout. Together with the observations of others, our findings suggest that the deacetylation activity of SIRT1 suppressed the function of NRF2 in driving an antiinflammatory program in astrocytes.

Among the molecules with altered expression in *Sirt1^–/–^* astrocytes in EAE mice, of particular interest is the upregulated expression of CHI3L1, SRXN1, and TRAIL. CHI3L1 expression has been associated with the immunomodulatory property of mesenchymal stem cells (55) and macrophages (43, 56). Expression of CHI3L1 in the CNS is predominantly associated with reactive astrocytes in the vicinity of inflammatory lesions, and CHI3L1-deficient mice show more severe EAE and increased immune cell infiltrates and gliosis in the CNS (42). Here, we showed enhanced CHI3L1 expression in *Sirt1^–/–^* astrocytes, which produced increased levels of IL-5, IL-10, and TGF-β, supporting the notion of an antiinflammatory function of CHI3L1 in reactive astrocytes in EAE. SRXN1, an endogenous antioxidant protein, exhibits neuroprotective effects, and loss of its expression in astrocytes may cause excessive activation of inflammatory responses and contribute to stress-induced neuronal death (44). These data suggest that upregulation of SRXN1 expression in astrocytes may, therefore, protect astrocyte-specific *Sirt1^–/–^* mice from EAE as shown in our study. Furthermore, researchers recently identified a novel subset of lysosomal membrane glycoprotein 1 (LAMP1) and TRAIL (LAMP1^+^TRAIL^+^) astrocytes that limit CNS inflammation by inducing T cell apoptosis through TRAIL/DR5 signaling (45, 57). Consistent with these important observations, our data showed enhanced expression of TRAIL on astrocytes from astrocyte-specific *Sirt1^–/–^* EAE mice, with reduced numbers of CD4^+^ T cells in the CNS. Thus, in astrocyte-specific *Sirt1^–/–^* EAE mice, enhanced TRAIL expression on astrocytes may be an important mechanism underlying the reduced numbers of CD4^+^ T cells and the inflammatory demyelination of the CNS in astrocyte-specific *Sirt1^–/–^* EAE mice.

Considerable progress has been made in immunomodulatory therapies that reduce the severity and progression of MS; however, existing therapies mainly target the peripheral immune system and have side effects such as suppression of systemic immune responses (58, 59). An approach to overcome this weakness could be targeting only the CNS, and astrocytes could be an ideal target for this purpose. Indeed, in astrocytes, blocking the signaling of proinflammatory molecules such as IL-17 ameliorated EAE (60, 61). EAE was also suppressed by inactivation of other proinflammatory molecules in astrocytes, including B4GALT6 (62), inositol-requiring enzyme-1α (IRE1α), X-box binding protein 1 (XBP1) (63), Ugcg (30), MAFG, MAT2A, and GM/CSF signaling (54), whereas CNS autoimmunity worsened by inactivation of immunomodulatory molecules such as AHR (64, 65) or NRF2 (54) in astrocytes. Consistent with these observations, in the present study, we verified the potential of SIRT1 inactivation in astrocytes as a method to treat ongoing disease using the CRISPR/Cas technique.

AAV has been safely used in clinical trials for neurological disorders, e.g., Parkinson’s disease (66). Using the CAS13-mediated RNA-targeting technique developed in recent years to silence *Sirt1* expression in astrocytes could provide a safe and feasible method for clinical use (67, 68). Furthermore, CNS-specific treatment directly targets the inflammatory demyelination process in the lesion foci and could thus be more effective than systemic treatments. For example, while systemic administration of IL-10 failed to suppress EAE, the delivery of cells that expressed IL-10 into the CNS had a significant therapeutic effect (69). Together, our findings demonstrate that *Sirt1* expression in reactive astrocytes played a pathogenic role in inflammatory demyelination, and its inactivation in these cells may represent a strategy for the treatment of CNS-specific EAE and MS.

## Methods

### Animals.

C57BL/6J (stock no. 000664), GFAP-Cre (stock no. 024098), *Sirt1^fl/fl^* (stock no. 029603), and LSL-Cas9 (stock no. 026175) mice were purchased from The Jackson Laboratory. Astrocyte-specific *Sirt1^–/–^* mice were generated by crossing GFAP-Cre and *Sirt1^fl/fl^* mice, and deletion of *Sirt1* in astrocytes was verified by PCR and Western blotting (data not shown). All animals were kept in a pathogen-free facility at Thomas Jefferson University.

### Cell lines.

The N2A-Cas9 cell line was purchased from Genecopoeia (Rockville) and grown in DMEM containing 10% FBS. HEK293 cells were also cultured in DMEM containing 10% FBS (Thermo Fisher Scientific). Cells were maintained at 37°C in a 5% CO_2_ atmosphere.

### Plasmids.

LentiCRISPR v2 and AAV:ITR-U6-sgRNA(backbone)-pCBh-Cre-WPRE-hGHpA-ITR were a gift from Feng Zhang (MIT, Cambridge, Massachusetts, USA). For the construction of pLenti-sgRNA(backbone)-EFS-Cre-P2A-Puro, the *Cas9* sequence in lentiCRISPR v2 was replaced by a *Cre* gene sequence (Supplemental Figure 1A). sgRNAs targeting mouse *Sirt1* or *Nfe2l2* were subcloned into pLenti- sgRNA(backbone)-EFS-Cre-P2A-Puro through *BsmB*; the obtained plasmids were named pLenti-EFS-Cre-P2A-Puro-sgSirt1 or pLenti-EFS-Cre-P2A-Puro-sgNfe2l2. Scrambled sgRNA (sgScram) was also subcloned into pLenti-EFS-Cre-P2A-Puro as a control.

To knock out *Sirt1* in adult mouse astrocytes, the GFAP promoter was amplified from the pLenti-Gfap-eGFP-mir30-shAct1 vector (61) and subcloned into AAV:ITR-U6-sgRNA(backbone)-pCBh-Cre-WPRE-hGHpA-ITR to replace the CBh promoter; the obtained plasmid was named pAAV-sgRNA(backbone)-GFAPp-Cre (Supplemental Figure 1B). The U6-sgSirt1 or U6-sgScram cassette was cleaved from pLenti-EFS-Cre-P2A-Puro-sgSirt1 or pLenti-EFS-Cre-P2A-Puro-sgScram and subcloned into pAAV-sgRNA(backbone)-GFAPp-Cre; the resulting plasmid was named pAAV-sgSirt1-GFAPp-Cre or pAAV-sgScram-GFAPp-Cre.

pAdδF6 was a gift from James M. Wilson (University of Pennsylvania, Philadelphia, Pennsylvania, USA); pUCmini-iCAP-PHP.eB was a gift from Viviana Gradinaru (California Institute of Technology, Pasadena, California, USA); psPAX2 and pMD2.G were gifts from Didier Trono (École Polytechnique Fédérale de Lausanne [EPFL], Lausanne, Switzerland). The primers used are listed in Supplemental Table 1.

### sgRNA design and screen.

sgRNAs targeting *Sirt1* and *Nfe2l2* were designed using the Benchling CRISPR design tool (https://www.benchling.com/crispr/); the corresponding primers were synthesized by Integrated DNA Technologies. Primers were annealed and ligated into pLenti-EFS-Cre-P2A-Puro, and sgRNA activity was analyzed in the N2A-Cas9 cell line. For sgRNA activity analysis, N2A-Cas9 cells were seeded in a 24-well plate at 1 × 10^5^ cells per well and, on the following day, were transfected with a mixture containing 0.5 μg sgRNA-carrying plasmid and 1 μL Lipofectamine 2000 (Thermo Fisher Scientific). The medium was changed on the next day, and cells were collected 48 hours after transfection. Knockout efficiency was analyzed by Western blotting. The primers used are listed in Supplemental Table 1.

### Virus packaging and purification.

The lentivirus particles were generated by transfecting 293T cells with the transfer plasmid, psPAX2, and pMD2.G using PEI-MAX (Polysciences). Supernatant was collected 30 hours and 48 hours after transfection, filtered through a 0.45 μm PVDF filter, and concentrated overnight with 40% PEG-10000 (MilliporeSigma).

The AAV particles were generated as reported by Chan et al. (38). Low-passaged 293T cells were transfected with the transfer plasmid, pUCmini-iCAP-PHP.eB, and pAdDeltaF6 using PEI-MAX (Polysciences); viral particles were collected from the medium 72 hours after transfection and from cells and the medium 120 hours after transfection. The supernatant was concentrated with 40% PEG-8000 (Thermo Fisher Scientific) and combined with cell pellets for processing. The cell pellets were suspended in 500 mM NaCl, 40 mM Tris, 2.5 mM MgCl_2_, pH 8, and 100 U/mL salt-activated nuclease (SAN) (MilliporeSigma) at 37°C for 1 hour. After this, the cell lysates were clarified by centrifugation at 2,000*g* and then purified using iodixanol (MilliporeSigma) step gradients (15%, 25%, 40%, and 60%) (70). Viruses were concentrated using Amicon filters (MilliporeSigma) and formulated in sterile PBS with 0.001% Pluronic-F68 (Thermo Fisher Scientific). Virus titers were measured by determining the number of DNAse I–resistant vector genomes (vg) using quantitative PCR (qPCR) with a linearized genome plasmid as a standard (71).

### EAE induction and treatment.

Female 8- to 10-week-old GFAP^Cre^ *Sirt1^fl/fl^* and *Sirt1^fl/fl^* mice were used for EAE induction. Mice were immunized subcutaneously at 2 sites on the back with 200 μg MOG_35–55_ peptide (GenScript) emulsified in Complete Freund’s Adjuvant (CFA) (BD Biosciences) supplemented with 4 mg/mL *Mycobacterium tuberculosis* H37Ra (BD Biosciences); 200 ng pertussis toxin (MilliporeSigma) was injected intraperitoneally into each mouse on day 0 and day 2 p.i. All mice were monitored for weight and clinical signs daily until 25 or 30 days after induction of EAE. Mice were euthanized if they showed a 20% loss of maximum body weight. Gel food was supplied at the onset of EAE disease. Clinical scores were recorded on the following scale: 0, no clinical signs; 1, limp tail; 2, limp tail with weak/partially paralyzed hind legs; 3, limp tail with completely paralyzed hind legs; 4, tetraplegia; 5, moribund.

### Astrocyte-specific Sirt1 knockout during ongoing EAE.

AAV PHP.eB at 2 × 10^11^ vg was diluted in 200 μL PBS with 0.001% Pluronic-F68, and then injected through the tail vein into LSL-Cas9 mice on day 15 p.i. Two weeks after injection, the mice were sacrificed, and knockout efficiency was analyzed by immunostaining.

### Isolation of immune cells from the CNS and spleen.

Brains and spinal cords from naive and EAE mice were removed, minced, and enzymatically dissociated with Liberase TL (Roche) for 30 minutes at 37°C. Liberase was neutralized by DMEM supplemented with 10% FBS. Cells were then passed through a 70 μm cell strainer and centrifuged, resuspended in 30% Percoll (MilliporeSigma), overlaid onto 70% Percoll, and centrifuged at 800*g* at 4°C for 20 minutes with slow acceleration and deceleration settings. Immune cells were collected from the 30%–70% interphase. Spleens from naive or EAE mice were dispersed to the single-cell level by passage through a 40 μm cell strainer, and erythrocytes were removed using RBC Lysis Buffer (BioLegend). For flow cytometric analysis, cells were seeded in a 24-well plate at a concentration of 1 × 10^6^ cells per well, treated with PMA (50 ng/mL; MilliporeSigma), ionomycin (500 ng/mL; MilliporeSigma), and BD GolgiPlug (1 μg/mL; BD Biosciences) for 4 hours and then analyzed by flow cytometry.

### Immune cell migration assay.

Immune cells were isolated from the spleens of naive or EAE mice and seeded in the upper chamber of a 24-well cell culture insert with a 5 μm pore size (Corning). The lower chamber was filled with culture supernatant of cocktail-stimulated *Sirt1*-knockout or WT astrocytes (ACM). Migrating immune cells were quantified in the lower chamber after 2 hours by cell counting and flow cytometry.

### Flow cytometry.

Cells were first stained with a surface antibody at 4°C for 20 minutes, fixed with Fixation Medium (Medium A, Thermo Fisher Scientific), washed, and then incubated with an intracellular antibody dissolved in Permeabilization Medium (Medium B, Thermo Fisher Scientific) at 4°C overnight. The following antibodies were used in this study: APC anti–mouse CD45 (clone 30-F11, BD Biosciences); P-blue anti–mouse CD4 (clone RM4-5, BD Biosciences); PE anti–mouse IL-17 (clone TC11-18H10, BD Biosciences); BV711 anti–mouse IFN-γ (clone XMG1.2, BD Biosciences); AF488 anti–mouse Foxp3 (clone FJK-16s, eBioscience); PerCP-Cy5.5 anti–mouse CD11b (clone M1/70, BD Biosciences); PE-Cy7 anti–mouse IL-4 (clone 11b11, BD Biosciences); BV605 anti–mouse IL-10 (clone JES5-16E3, BioLegend); and PE-Dazzle594 anti-TNF (clone, MP6-XT22, BioLegend). Compensation was performed with UltraComp eBeads (01-2222-42, Thermo Fisher Scientific).

### Isolation of primary astrocytes.

Primary astrocytes were isolated as previously reported (54). Cerebral cortices of P0–P3 mice were dissected, carefully stripped of their meninges, and digested with 2 U/mL Liberase TL (Roche) at 37°C for 15 minutes. Liberase was neutralized by DMEM supplemented with 10% FBS, and cells were passed through a 70 μm cell strainer. The cell suspension was then cultured with DMEM containing 10% FBS at 37°C in humidified 5% CO_2_ and 95% air in T-175 cell culture flasks for 7–10 days until confluence. Medium was replaced every 4–5 days. After the cells reached confluence, microglia and oligodendrocytes were removed by shaking the glia culture at 260 rpm at 37°C overnight and washing extensively with PBS; the remaining attached cells were astrocytes with greater than 98% purity (Supplemental Figure 2). It has been shown that primary astrocytes cultured with serum-containing medium may induce a reactive phenotype in astrocytes (72). Although FBS-containing medium was used during astrocyte isolation to enhance their viability, these cells were cultured in a serum-free condition. These cells did not express reactive astrocyte markers in the nonstimulated culture condition (as shown in Figure 1H), indicating a minor effect of FBS during astrocyte isolation, while culturing them in the serum-free condition.

### Astrocyte treatment in vitro.

For SIRT1 activation, 5×10^5^ primary astrocytes were seeded in a 6-well plate in astrocyte serum-free medium. The following day, the cells were incubated with 10 μg/mL resveratrol (MilliporeSigma) for 24 hours and then treated with a cytokine cocktail (C1q, IL-1α, TNF) for an additional 24 hours, as previously reported (8).

For *Sirt1*-knockout analysis, 5 × 10^5^ *Sirt1*-knockout or WT astrocytes were seeded in a 6-well plate in astrocyte serum-free medium. The following day, cells were treated with a cytokine cocktail for 24 hours, as previously reported (8).

For RT-PCR and ELISA analyses, the cells and supernatants were collected immediately after cocktail stimulation. For ACM collection, the cells, after cocktail stimulation, were washed with PBS and incubated with fresh, serum-free medium for an additional 24 hours. The supernatants were collected, filtered through a 0.45 μm filter, and kept at 4°C until use.

### Microarray.

Primary astrocytes isolated from P2–P3 LSL-Cas9 pups were infected with a sgSirt1- or sgScram-carrying lentivirus and then selected with 4 μg/mL puromycin for 3 days. Cells were stimulated with cocktail (C1q, IL-1α, TNF) for an additional 24 hours and then collected for microarray analysis. Four samples, namely sgScram-1, sgScram-2, sgSirt1-1, and sgSirt1-2, were analyzed at the Cancer Genomics and Bioinformatics Resource (CGBR), Thomas Jefferson University. The data have been deposited in NCBI’s Gene Expression Omnibus (GEO) database (GEO GSE212924).

### Sirt1 or Nfe2l2 knockout in vitro.

For knockout of *Sirt1* or *Nfe2l2*, primary mouse astrocytes isolated from LSL-Cas9 mice were incubated for 24 hours with *Sirt1* or *Nfe2l2* sgRNA-carrying lentiviruses and 8 μg/mL polybrene (MilliporeSigma), after which the medium was changed. Forty-eight hours after infection, cells were selected with 4 μg/mL puromycin for 3 days. Knockout of *Sirt1* or *Nfe2l2* was verified by Western blotting.

### Microglia isolation, culture, and treatment in vitro.

Brains from P7 mouse pups were dissociated into a single-cell suspension using the Neural Tissue Dissociation Kit (P) (Miltenyi Biotec) according to the manufacturer’s protocol. Microglia were isolated from the single-cell suspension using CD11b microbeads (Miltenyi Biotec) and cultured in DMEM/F12 with 10% FBS (73). Cells were stimulated with LPS (100 ng/mL) for 18 hours and then washed and incubated with fresh medium containing TGF-β or ACMs.

### OPC isolation and differentiation in vitro.

Brains from P2–P3 mouse pups were dissociated into a single-cell suspension using the Neural Tissue Dissociation Kit (P) (Miltenyi Biotec) according to the manufacturer’s protocol. OPCs were isolated from the single-cell suspension using CD140a microbeads (Miltenyi Biotec). OPC proliferation and differentiation media were prepared as previously described with some modifications (74). OPC proliferation medium comprised DMEM/F12 (Thermo Fisher Scientific), N2 Supplement (Thermo Fisher Scientific), B27 Supplement (Thermo Fisher Scientific), 20 ng/mL basic FGF (bFGF) (Peprotech), and 20 ng/mL PDGF-AA (Peprotech). bFGF and PDGF-AA were removed, and T3 (MilliporeSigma) was added in OPC differentiation medium. OPCs (2 × 10^3^) were seeded on presterilized glass coverslips (Carolina) coated with poly-d-lysine and laminin (both from MilliporeSigma) in a 24-well plate. Cells were kept in OPC proliferation medium for 2 days, and then in medium consisting of OPC differentiation medium and ACM at a ratio of 1:1 for 8 days. The medium was half-changed every 2–3 days. Differentiation was analyzed by cell immunostaining.

### IHC analysis.

Both mouse and human tissues were fixed with 4% paraformaldehyde, embedded in paraffin and cut into 4 μm sections. Paraffin sections were stained with H&E for assessment of inflammation and Luxol fast blue (LFB) for assessment of demyelination. For immunofluorescence staining, paraffin sections were deparaffinized, washed in running water, and treated with heat retrieval solution (Biocare). The slides were then cooled under running water, washed with TBS, permeated by TBS with 0.2% Triton X-100, and blocked in TBS with 10% horse serum and 1% BSA for 1 hour. The primary antibodies were then incubated in TBS with 1% horse serum and 1% BSA at 4°C overnight. The following day, the slides were washed 3 times in TBS with 0.025% Triton X-100 and then incubated with a secondary antibody (Jackson ImmunoResearch) in TBS with 1% horse serum and 1% BSA at room temperature for 1 hour. Frozen tissues were cut into 10 μm sections in our laboratory. The frozen sections were air dried, rehydrated in TBS, permeated by TBS with 0.2% Triton X-100, and blocked in TBS with 10% horse serum and 1% BSA for 30 minutes. The primary and secondary antibodies were then incubated as described above. Cells seeded on glass coverslips were washed with TBS, fixed by 4% paraformaldehyde, and permeated by TBS with 0.2% Triton X-100. Cells were then incubated with a primary antibody in TBS with 10% horse serum and 1% BSA for 1 hour at room temperature, washed in TBS 3 times, and incubated with a secondary antibody in TBS with 1% horse serum and 1% BSA for 30 minutes at room temperature. Finally, all of the sections and coverslips were washed and mounted in ProLong Gold Antifade Reagent with DAPI (Thermo Fisher Scientific). Imaging was performed using a Nikon A1R microscope and Nikon NIS Elements acquisition and analysis software. Images were processed and analyzed using ImageJ (NIH).

The following antibodies were used: goat anti-GFAP (mouse and human) (ab53554, Abcam); rabbit anti-GFAP (mouse and human) (12389, clone D1F4Q, Cell Signaling Technology); rabbit anti-mSIRT1 (ab12193, Abcam); mouse anti-mCNP (ab6319, clone 11-5B, Abcam); mouse anti-mAPC (OP80, clone CC1, EMD Millipore); goat anti-C3d (mouse and human) (AF2655, R&D Systems); rabbit anti-S100A10 (mouse and human) (MA5-15326, 4E7E10, Abcam); rabbit anti-SRXN1 (MBS716745, MyBioSource); rabbit anti-CHI3L1 (ab255297, EPR19078-157, Abcam); rabbit anti-TRAIL (ab231265, Abcam); chicken anti-GFP (ab13970, Abcam); rabbit anti-mNRF2 (NBP1-32822, Novus Biologicals); and mouse anti-hSIRT1 (04-1557; clone 10E4, EMD Millipore).

### NRF2 acetylation detection.

sgSirt1- or sgScram-treated astrocytes (3 × 10^6^) were seeded in a 100 mm dish, stimulated with cocktail for 24 hours, and then collected and lysed with 300 μL 1× cell lysis buffer (Cell Signaling Technology). Cell lysate (200 μL) was incubated with 2 μL anti-NRF2 antibody (NBP1-32822, Novus Biologicals) at 4°C overnight and then incubated with 20 μL protein A (Thermo Fisher Scientific) at 4°C for 3 hours. The beads were washed 3 times with 1× cell lysis buffer and centrifuged, and 50 μL 2 × loading buffer was added. The mixture was boiled at 95°C for 5 minutes to denature the proteins and dissociate them from the protein A beads. Then, they were centrifuged, and the supernatants were separated by SDS-PAGE and detected by purified anti–acetylated lysine antibody (623402, clone 15G10, BioLegend).

### Western blot analysis.

WT, *Sirt1-*knockout, or *Nfe2l2*-knockout astrocytes (1 × 10^6^) were lysed in 200 μL RIPA lysis buffer (Thermo Fisher Scientific) containing proteases inhibitors (MilliporeSigma). Cells were incubated on ice for 30 minutes and sonicated for 10 seconds, with the cells being kept on ice during sonication. The cell lysate was centrifuged for 10 minutes at 4°C. Protein concentrations were determined using the BCA Protein Assay Kit (Thermo Fisher Scientific). Protein lysates were diluted in SDS-PAGE sample buffer, separated on Novex 4%–12% Tris-Glycine gel (Thermo Fisher Scientific), and analyzed by Western blotting using rabbit anti-SIRT1 polyclonal antibody (Abcam) or anti-NRF2 antibody (Invitrogen, Thermo Fisher Scientific). GAPDH was detected with a rabbit anti-GAPDH monoclonal antibody (Cell Signaling Technology) and used as a loading control.

### RT-PCR.

RNA was extracted using the RNeasy Mini Kit (QIAGEN). cDNA was reverse-transcribed using QuantiTect Rev. Transcription Kit (QIAGEN). Gene expression was quantified by qPCR using SYBR Green Mix (Thermo Fisher Scientific). The expression of each gene was normalized to GAPDH and then to the control group. The primers used for this work are listed in Supplemental Table 1.

### MS tissues.

Brain tissue was obtained from untreated individuals with clinically diagnosed and neuropathologically confirmed MS (Rocky Mountain MS Center Tissue Bank, Aurora, Colorado, USA).

### Statistics.

Statistical analyses were performed with GraphPad Prism (GraphPad Software). An unpaired, 2-tailed *t* test was used for comparison of 2 groups. One-way ANOVA was applied for comparison of more than 2 groups. Two-way, repeated-measures ANOVA was used for comparison of clinical scores. *P* values of less than 0.05 were considered significant. All error bars represent the SEM or SD as noted in the figure legends. Unless otherwise stated, 3 or more independent experiments were used for all assays, and the figures show representative examples.

### Study approval.

All animal experiments were approved by the IACUC of Thomas Jefferson University (approval S25801). All individuals with MS or their next of kin had given informed consent for an autopsy and the use of their brain tissue for research purposes. All procedures have been approved by IRB of Thomas Jefferson University.

## Author contributions

WZ, DX, and GXZ conceived and designed the experiments, analyzed data, and wrote the manuscript. WZ and DX carried out the experiments. XL and YZ helped with the experimental design and statistical analysis. JR performed flow cytometry experiments. GC helped with the OPC differentiation experiments. AB helped with some of the in vitro experiments. DH, LL, WI, and RT helped with EAE experiments and revised the manuscript. MTC helped evaluate the immunohistological results and revised the manuscript. BC and AR co-supervised the study and wrote the manuscript. All authors read and approved the final manuscript.

## Supplementary Material

Supplemental data

## Version 1 09/22/2022

In-Press Preview

## Version 2 11/15/2022

Electronic publication

## References

[1] Brambilla R (2019). The contribution of astrocytes to the neuroinflammatory response in multiple sclerosis and experimental autoimmune encephalomyelitis. Acta Neuropathol.

[2] Giovannoni F, Quintana FJ (2020). The role of astrocytes in CNS inflammation. Trends Immunol.

[3] Lee HG (2022). Function and therapeutic value of astrocytes in neurological diseases. Nat Rev Drug Discov.

[4] Anderson MA (2016). Astrocyte scar formation aids central nervous system axon regeneration. Nature.

[5] Nash B (2011). Functional duality of astrocytes in myelination. J Neurosci.

[6] Fulmer CG (2014). Astrocyte-derived BDNF supports myelin protein synthesis after cuprizone-induced demyelination. J Neurosci.

[7] Pitt D, Ponath G (2019). Astrocytes play a crucial role in the formation and evolution of MS lesions - Yes. Mult Scler.

[8] Liddelow SA (2017). Neurotoxic reactive astrocytes are induced by activated microglia. Nature.

[9] Escartin C (2021). Reactive astrocyte nomenclature, definitions, and future directions. Nat Neurosci.

[10] Chang HC, Guarente L (2014). SIRT1 and other sirtuins in metabolism. Trends Endocrinol Metab.

[11] Haigis MC, Sinclair DA (2010). Mammalian sirtuins: biological insights and disease relevance. Annu Rev Pathol.

[12] Zhang J (2009). The type III histone deacetylase Sirt1 is essential for maintenance of T cell tolerance in mice. J Clin Invest.

[13] Gao B (2012). Analysis of sirtuin 1 expression reveals a molecular explanation of IL-2-mediated reversal of T-cell tolerance. Proc Natl Acad Sci U S A.

[14] Kwon HS (2012). Three novel acetylation sites in the Foxp3 transcription factor regulate the suppressive activity of regulatory T cells. J Immunol.

[15] Liu G (2015). Dendritic cell SIRT1-HIF1α axis programs the differentiation of CD4+ T cells through IL-12 and TGF-β1. Proc Natl Acad Sci U S A.

[16] van Loosdregt J (2010). Regulation of Treg functionality by acetylation-mediated Foxp3 protein stabilization. Blood.

[17] Beier UH (2011). Histone/protein deacetylases control Foxp3 expression and the heat shock response of T-regulatory cells. Curr Opin Immunol.

[18] Daenthanasanmak A (2019). Targeting Sirt-1 controls GVHD by inhibiting T-cell allo-response and promoting Treg stability in mice. Blood.

[19] Lim HW (2015). SIRT1 deacetylates RORgammat and enhances Th17 cell generation. J Exp Med.

[20] Yang H (2013). Histone deacetylase sirtuin 1 deacetylates IRF1 protein and programs dendritic cells to control Th17 protein differentiation during autoimmune inflammation. J Biol Chem.

[21] Di Sante G (2015). Sirt1-deficient mice have hypogonadotropic hypogonadism due to defective GnRH neuronal migration. Mol Endocrinol.

[22] Zhang Z (2019). Sirt1 improves functional recovery by regulating autophagy of astrocyte and neuron after brain injury. Brain Res Bull.

[23] Pallas M (2008). Modulation of sirtuins: new targets for antiageing. Recent Pat CNS Drug Discov.

[24] Rafalski VA (2013). Expansion of oligodendrocyte progenitor cells following SIRT1 inactivation in the adult brain. Nat Cell Biol.

[25] Prozorovski T (2019). Regulation of sirtuin expression in autoimmune neuroinflammation: induction of SIRT1 in oligodendrocyte progenitor cells. Neurosci Lett.

[26] Li D (2017). Interactions between Sirt1 and MAPKs regulate astrocyte activation induced by brain injury in vitro and in vivo. J Neuroinflammation.

[27] Cheng Y (2014). Sirtuin 1 attenuates oxidative stress via upregulation of superoxide dismutase 2 and catalase in astrocytes. J Neuroimmunol.

[28] Aguirre-Rueda D (2015). Astrocytes protect neurons from Aβ1-42 peptide-induced neurotoxicity increasing TFAM and PGC-1 and decreasing PPAR-γ and SIRT-1. Int J Med Sci.

[29] Prozorovski T (2008). Sirt1 contributes critically to the redox-dependent fate of neural progenitors. Nat Cell Biol.

[30] Chao CC (2019). Metabolic control of astrocyte pathogenic activity via cPLA2-MAVS. Cell.

[31] Platt RJ (2014). CRISPR-Cas9 knockin mice for genome editing and cancer modeling. Cell.

[32] Zhou B (2019). Astrocyte morphology: diversity, plasticity, and role in neurological diseases. CNS Neurosci Ther.

[33] Lepennetier G (2019). Cytokine and immune cell profiling in the cerebrospinal fluid of patients with neuro-inflammatory diseases. J Neuroinflammation.

[34] Noh MY (2016). Mesenchymal stem cells modulate the functional properties of microglia via TGF-β secretion. Stem Cells Transl Med.

[35] Hayes JD, Dinkova-Kostova AT (2014). The Nrf2 regulatory network provides an interface between redox and intermediary metabolism. Trends Biochem Sci.

[36] Tu W (2019). The anti-inflammatory and anti-oxidant mechanisms of the Keap1/Nrf2/ARE signaling pathway in chronic diseases. Aging Dis.

[37] Kawai Y (2011). Acetylation-deacetylation of the transcription factor Nrf2 (nuclear factor erythroid 2-related factor 2) regulates its transcriptional activity and nucleocytoplasmic localization. J Biol Chem.

[38] Chan KY (2017). Engineered AAVs for efficient noninvasive gene delivery to the central and peripheral nervous systems. Nat Neurosci.

[39] Xiao D (2021). CRISPR-mediated rapid generation of neural cell-specific knockout mice facilitates research in neurophysiology and pathology. Mol Ther Methods Clin Dev.

[40] Keeler GD (2018). Gene therapy-induced antigen-specific Tregs inhibit neuro-inflammation and reverse disease in a mouse model of multiple sclerosis. Mol Ther.

[41] Henrik Heiland D (2019). Tumor-associated reactive astrocytes aid the evolution of immunosuppressive environment in glioblastoma. Nat Commun.

[42] Bonneh-Barkay D (2012). Exacerbation of experimental autoimmune encephalomyelitis in the absence of breast regression protein 39/chitinase 3-like 1. J Neuropathol Exp Neurol.

[43] He CH (2013). Chitinase 3-like 1 regulates cellular and tissue responses via IL-13 receptor α2. Cell Rep.

[44] Yu S (2015). Sulfiredoxin-1 protects primary cultured astrocytes from ischemia-induced damage. Neurochem Int.

[45] Sanmarco LM (2021). Gut-licensed IFNγ^+^ NK cells drive LAMP1^+^TRAIL^+^ anti-inflammatory astrocytes. Nature.

[46] Jablonska B (2016). Sirt1 regulates glial progenitor proliferation and regeneration in white matter after neonatal brain injury. Nat Commun.

[47] Assefa EG (2019). Role of resveratrol on indoxyl sulfate-induced endothelial hyperpermeability via aryl hydrocarbon receptor (AHR)/Src-dependent pathway. Oxid Med Cell Longev.

[48] Alesci A (2022). Resveratrol and immune cells: a link to improve human health. Molecules.

[49] Ren Z (2019). The role of different SIRT1-mediated signaling pathways in toxic injury. Cell Mol Biol Lett.

[50] Cuadrado A (2019). Therapeutic targeting of the NRF2 and KEAP1 partnership in chronic diseases. Nat Rev Drug Discov.

[51] Johnson DA (2010). The absence of the pro-antioxidant transcription factor Nrf2 exacerbates experimental autoimmune encephalomyelitis. Toxicol Sci.

[52] Bernstein AI, Miller GW (2010). Oxidative signaling in experimental autoimmune encephalomyelitis. Toxicol Sci.

[53] Hammer A (2017). Role of nuclear factor (erythroid-derived 2)-like 2 signaling for effects of fumaric acid esters on dendritic cells. Front Immunol.

[54] Wheeler MA (2020). MAFG-driven astrocytes promote CNS inflammation. Nature.

[55] Liu Q (2021). Mesenchymal stem cells alleviate experimental immune-mediated liver injury via chitinase 3-like protein 1-mediated T cell suppression. Cell Death Dis.

[56] Chen A (2021). Chitinase-3-like 1 protein complexes modulate macrophage-mediated immune suppression in glioblastoma. J Clin Invest.

[57] Kwon AHK, Liddelow SA (2021). Astrocytes have a license to kill inflammatory T cells. Immunity.

[58] Filippi M (2020). Identifying progression in multiple sclerosis: new perspectives. Ann Neurol.

[59] Reich DS (2018). Multiple sclerosis. N Engl J Med.

[60] Kang Z (2010). Astrocyte-restricted ablation of interleukin-17-induced Act1-mediated signaling ameliorates autoimmune encephalomyelitis. Immunity.

[61] Yan Y (2012). CNS-specific therapy for ongoing EAE by silencing IL-17 pathway in astrocytes. Mol Ther.

[62] Mayo L (2014). Regulation of astrocyte activation by glycolipids drives chronic CNS inflammation. Nat Med.

[63] Wheeler MA (2019). Environmental control of astrocyte pathogenic activities in CNS inflammation. Cell.

[64] Rothhammer V (2016). Type I interferons and microbial metabolites of tryptophan modulate astrocyte activity and central nervous system inflammation via the aryl hydrocarbon receptor. Nat Med.

[65] Rothhammer V (2018). Microglial control of astrocytes in response to microbial metabolites. Nature.

[66] Singh A, Sen D (2016). Therapeutic value of adeno associated virus as a gene therapy vector for parkinson’s disease — a focused review. Curr Gene Ther.

[67] Abudayyeh OO (2017). RNA targeting with CRISPR-Cas13. Nature.

[68] Konermann S (2018). Transcriptome engineering with RNA-targeting type VI-D CRISPR effectors. Cell.

[69] Croxford JL (2001). Different therapeutic outcomes in experimental allergic encephalomyelitis dependent upon the mode of delivery of IL-10: a comparison of the effects of protein, adenoviral or retroviral IL-10 delivery into the central nervous system. J Immunol.

[70] Zolotukhin S (1999). Recombinant adeno-associated virus purification using novel methods improves infectious titer and yield. Gene Ther.

[71] Gray SJ (2011). Production of recombinant adeno-associated viral vectors and use in in vitro and in vivo administration. Curr Protoc Neurosci.

[72] Prah J (2019). A novel serum free primary astrocyte culture method that mimic quiescent astrocyte phenotype. J Neurosci Methods.

[73] Butovsky O (2014). Identification of a unique TGF-β-dependent molecular and functional signature in microglia. Nat Neurosci.

[74] Najm FJ (2013). Transcription factor-mediated reprogramming of fibroblasts to expandable, myelinogenic oligodendrocyte progenitor cells. Nat Biotechnol.

