# Supplementary material for: SIRT1 inactivation switches reactive astrocytes to an antiinflammatory phenotype in CNS autoimmunity
Source: J Clin Invest. 2022 Nov 15;132(22):e151803. doi: 10.1172/JCI151803 (PMC9663155; doi:10.1172/JCI151803)
Supplement: Supplemental data [file jci-132-151803-s052.pdf]

Supplemental Data

SIRT1 inactivation switches reactive astrocytes to an anti-inflammatory phenotype in CNS autoimmunity

Weifeng Zhang, Dan Xiao, Xing Li, Yuan Zhang, Javad Rasouli, Giacomo Casella, Alexandra Boehm, Daniel Hwang, Larissa Lumi Watanabe Ishikawa, Rodolfo Thome, Bogoljub Ciric, Mark T. Curtis, Abdolmohamad Rostami, Guang-Xian Zhang

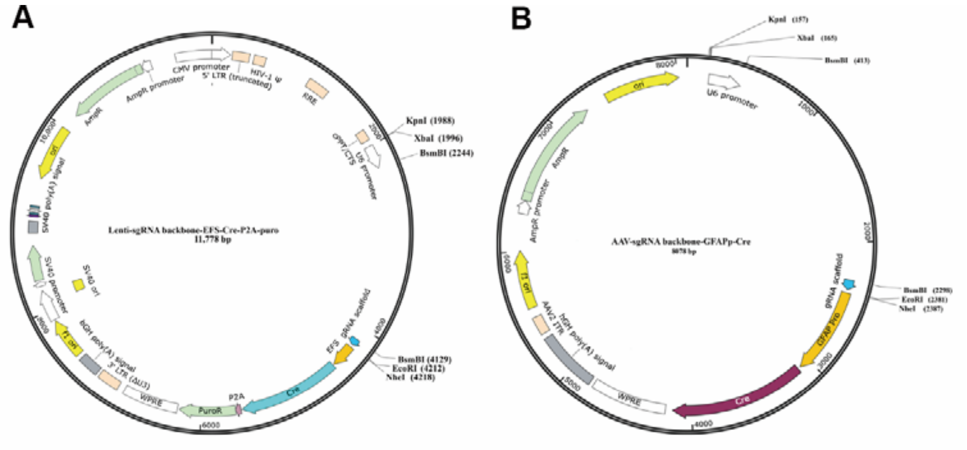

Supplemental Figure 1. Structure of plasmids. (A) Structure of CRISPR carrying lentivirus transfer plasmid for knockout. **(B)** Structure of CRISPR carrying AAV transfer plasmid for knockout in vivo.

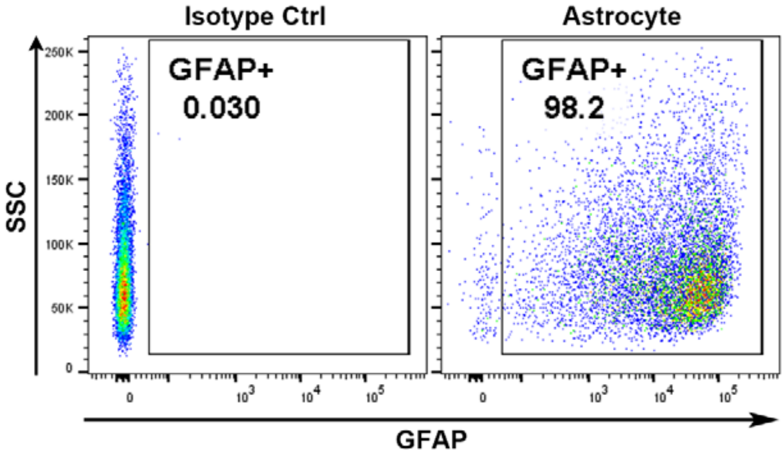

Supplemental Figure 2. Purity of primary astrocytes. Astrocytes were isolated from brains of C57BL/6 pups (P2) as described in "Materials and Methods," and their purity was analyzed by flow cytometry.

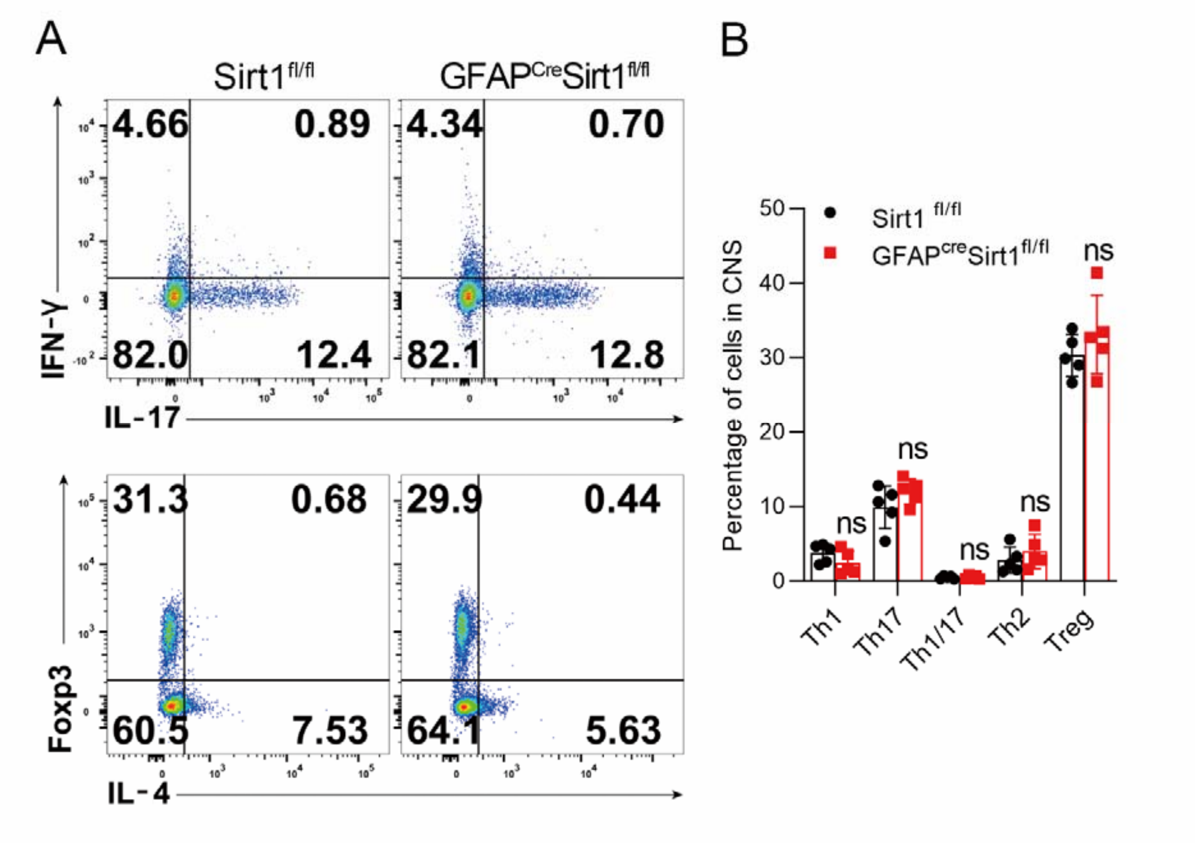

Supplemental Figure 3. Flow cytometry analysis of percentages of different Th subtypes in the CNS of Sirt1$^{fl/fl}$ and GFAP^{cre}Sirt1$^{fl/fl}$ EAE mice. EAE was induced in Sirt1$^{fl/fl}$ and GFAP^{cre}Sirt1$^{fl/fl}$ mice by MOG$_{35\text{-}55}$ peptide in CFA and pertussis toxin; mice were sacrificed at 25 days p.i., and MNCs were isolated from CNS tissues. **(A)** Representative image of flow cytometry result. **(B)** Statistical analysis of **(A)**. n=5 mice per group, unpaired two-tailed t test.

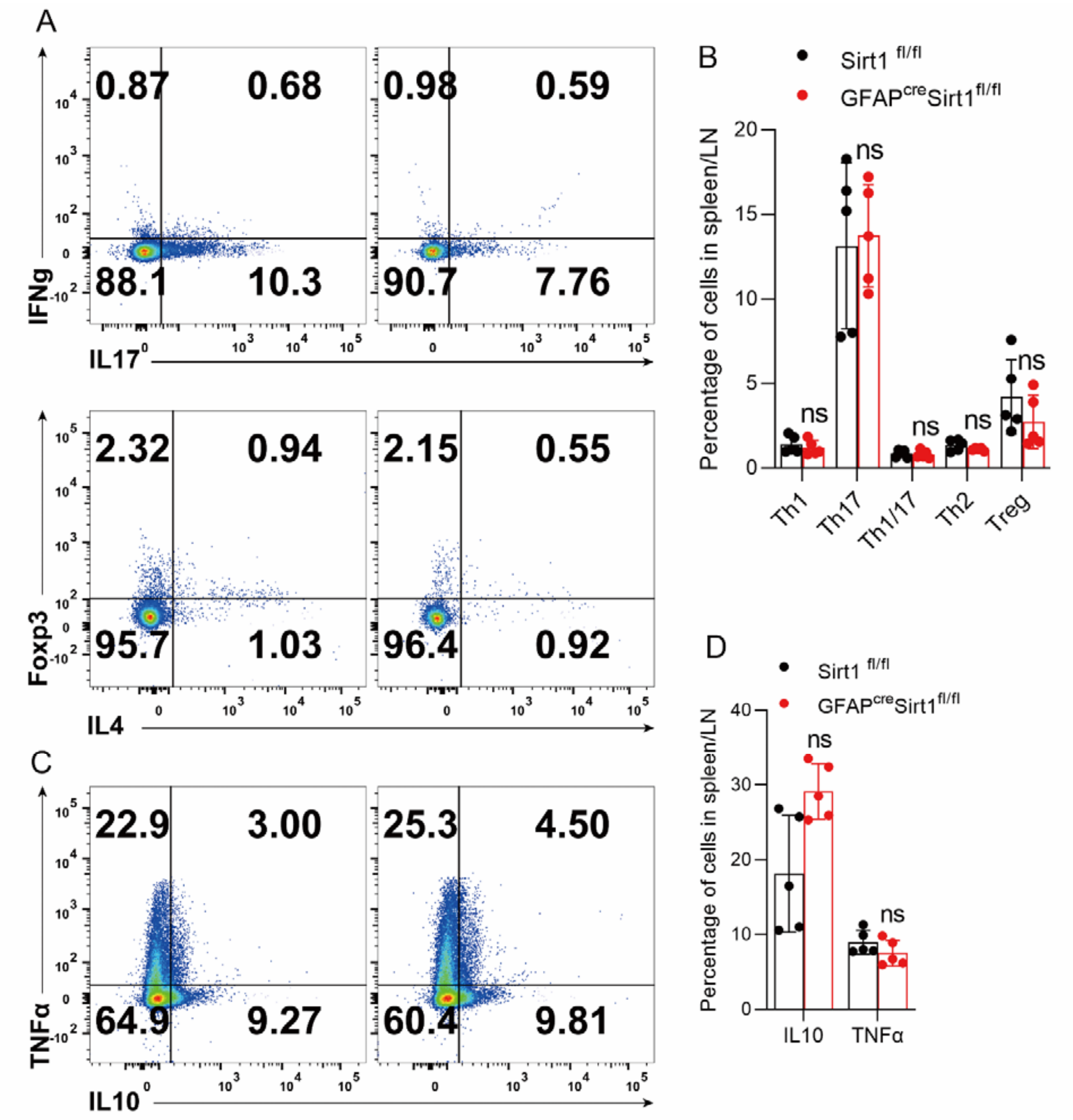

Supplemental Figure 4. Flow cytometry analysis of splenocytes of Sirt1$^{fl/fl}$ and GFAP^{cre}Sirt1$^{fl/fl}$ EAE mice. Splenocytes were harvested from EAE mice at day 25 p.i. **(A)** Flow cytometry analysis of different subtypes of Th cells. **(B)** Statistical analysis of (**A**). **(C)** Flow cytometry analysis of myeloid cells. **(D)** Statistical analysis of c. n=5 mice per group, unpaired two-tailed t test.

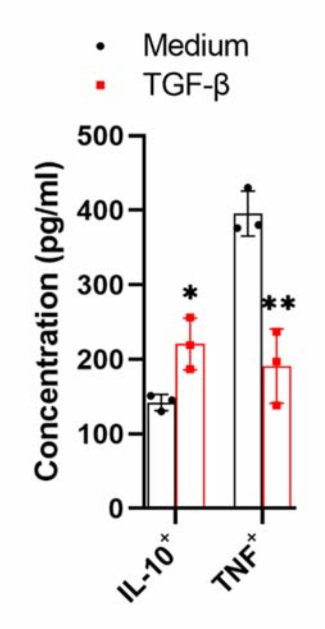

Supplemental Figure 5. ELISA analysis of the effects of TGF-β on microglia. Microglia were isolated from brains of newborn WT mice, as described in "Materials and Methods," stimulated with LPS for 18 h, and treated with TGF-β or medium for 24 h. Cells were then washed and cultured in fresh medium for additional 24 h, and the concentrations of TNF and IL-10 in culture supernatants were measured by ELISA. n = 3 samples per group, unpaired two-tailed t test, *$p<0.05$, **$p<0.01$.

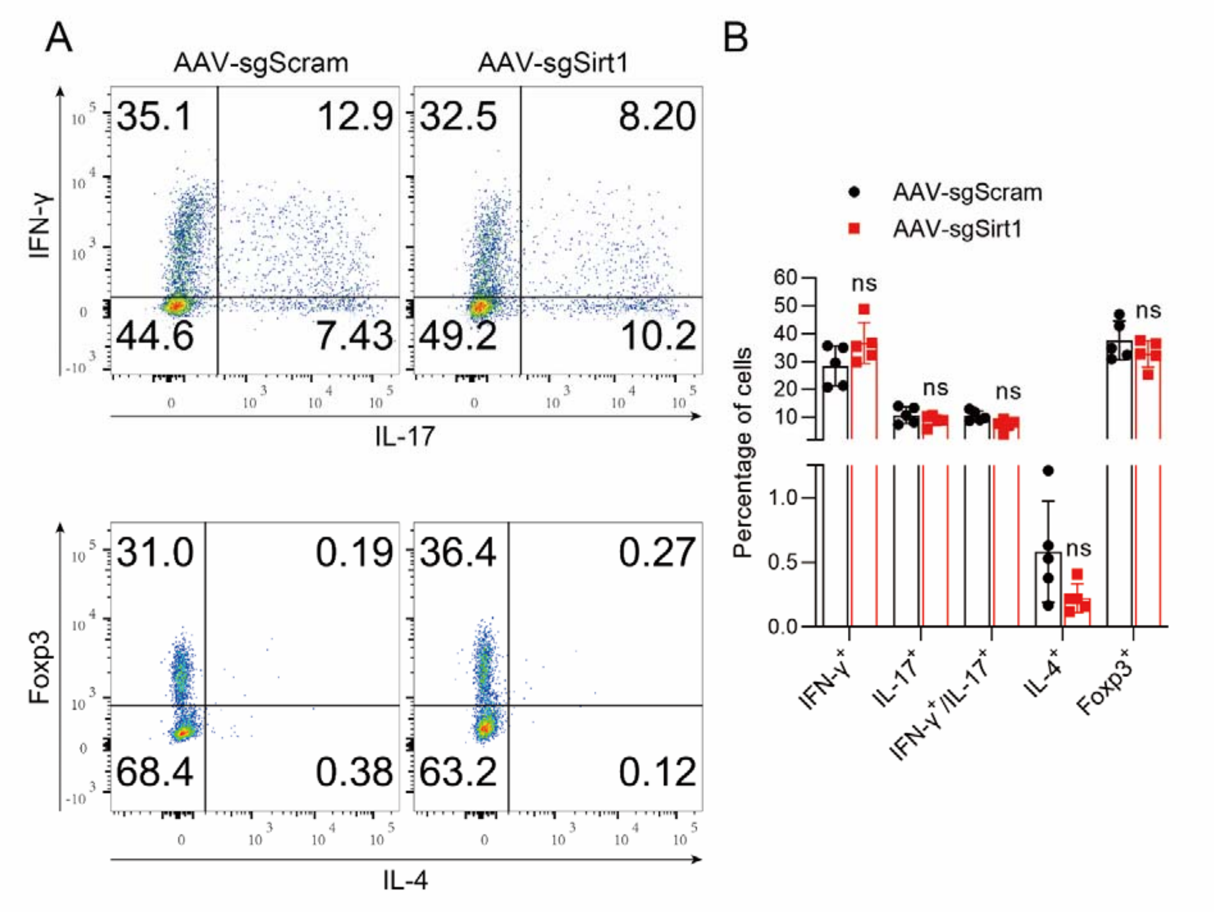

Supplemental Figure 6. Flow cytometry analysis of the percentages of different subtypes of Th cells in the CNS of AAV-sgScram- and AAV-sgSirt1-injected EAE mice. EAE was induced in LSL-Cas9 mice by MOG_{35-55} peptide in CFA and pertussis toxin. AAV-sgScram or AAV-sgSirt1 was injected through the tail vein at day 15 p.i. Mice were sacrificed at day 30 p.i. and MNCs were isolated from CNS tissues. **(A)** Representative image of flow cytometry analysis. **(B)** Statistical analysis of (**A**). n=5 mice per group, unpaired two-tailed t test.

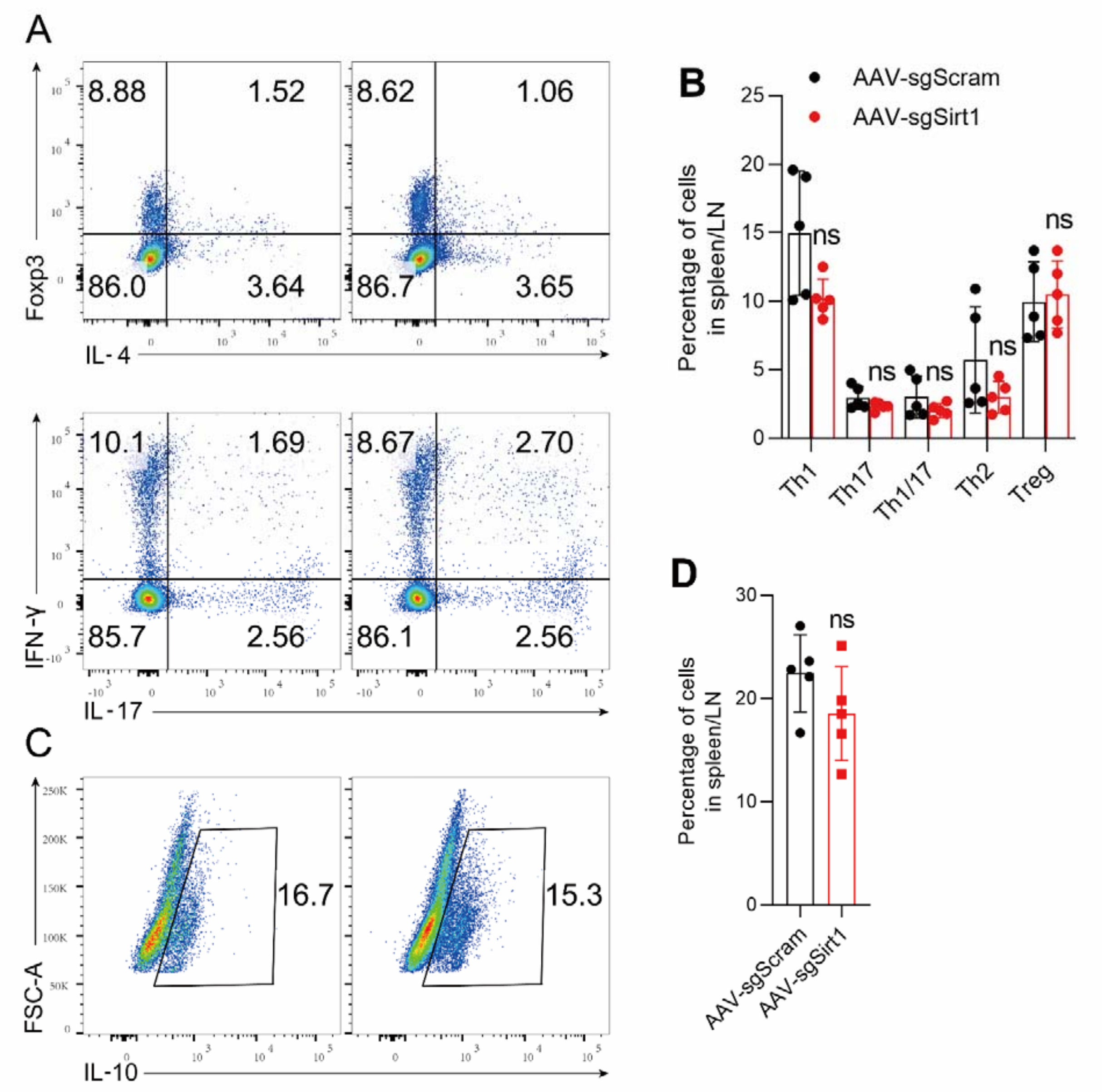

Supplemental Figure 7. Flow cytometry analysis of splenocytes of AAV-sgScram and AAV-sgSirt1 injected EAE mice. Splenocytes were harvested from AAV-Scram or AAV-*Sirt1* injected EAE mice at day 30 p.i. **(A)** Flow cytometry analysis of different subtypes of Th cells. **(B)** Statistical analysis. **(C)** Flow cytometry analysis of myeloid cells. **(D)** Statistical analysis of **(C)**. B and D, n=5 mice per group, unpaired two-tailed t test.

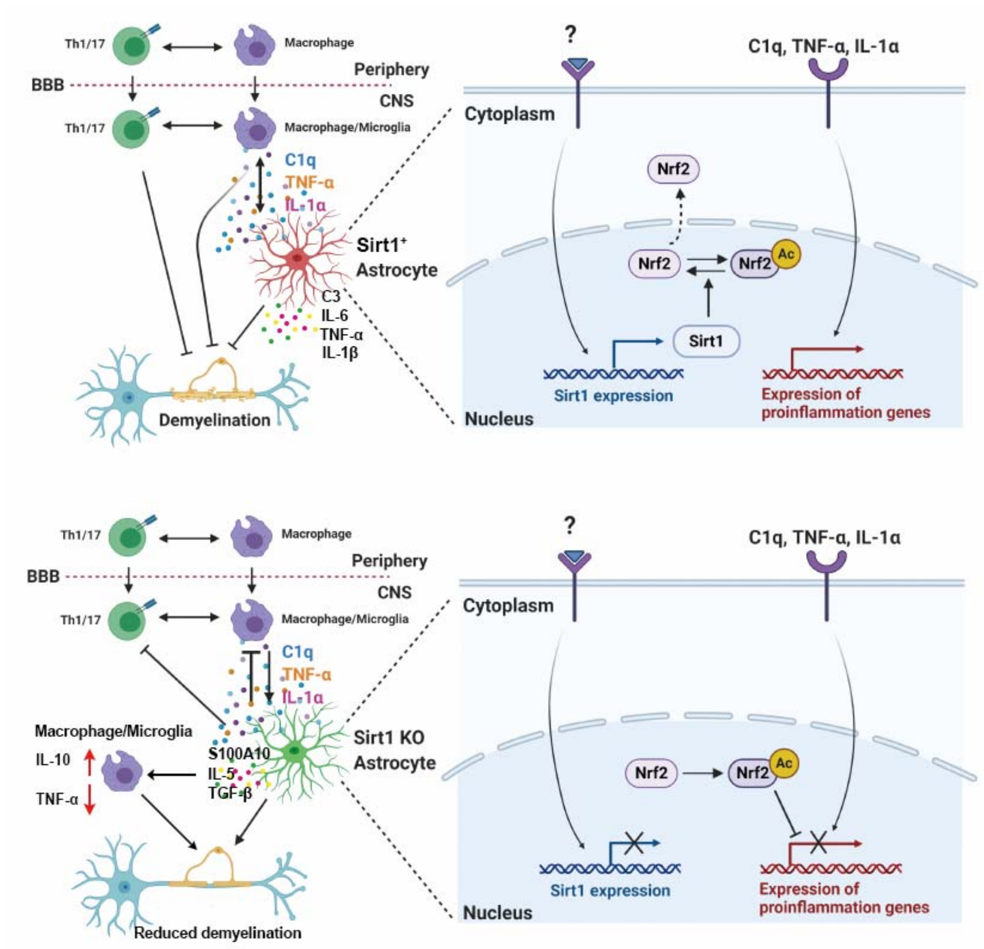

Supplementary Figure 8. Mechanism underlying SIRT1 enhancement of proinflammatory astrocytes and worsening of EAE. Myelin-reactive Th1/Th17 cells and macrophages, upon activation in the periphery, migrate into the CNS. Activated macrophages/microglia produce cytokines (C1q, TNF, IL-1α) that induce proinflammatory/neurotoxic astrocytes, which promoted proinflammatory macrophage/microglia, enhanced infiltration of immune cells, and inhibited OPC differentiation. In contrast, inactivation of SIRT1 endowed these cells with an anti-inflammatory status, which inhibited the production of proinflammatory mediators of myeloid cells/microglia and promoted oligodendrocyte maturation. Mice with astrocyte-specific *Sirt1* knockout had suppressed progression of EAE, with reduced demyelination, decreased numbers of T cells, and increased rate of IL-10-producing macrophages/microglia of the CNS, whereas peripheral immune response remained unaffected. $Sirt1^{-/-}$

astrocytes expressed a range of target genes of NRF2, whose deficiency shifted the beneficial action of *Sirt1*$^{-/-}$ astrocytes into a detrimental one, indicating that enhanced NRF2 activity is an important mechanism underlying the anti-inflammatory property of astrocytes upon SIRT1 inactivation.

Supplemental Table 1. List of primers used in this paper

Name of primers	Sequence of primers
Aspg realtime for	GCTGCTGGCCATTTACACTG
Aspg realtime reverse	GTGGGCCTGTGCATACTCTT
Cd44 realtime for	ACCTTGGCCACCACTCCTAA
Cd44 realtime reverse	GCAGTAGGCTGAAGGGTTGT
Cp realtime for	TGTGATGGGAATGGGCAATGA
Cp realtime reverse	AGTGTATAGAGGATGTTCCAGGTCA
Cxcl10 realtime for	CCCACGTGTTGAGATCATTG
Cxcl10 realtime reverse	CACTGGGTAAAGGGGAGTGA
Gfap realtime for	AGAAAGGTTGAATCGCTGGA
Gfap realtime reverse	CGGCGATAGTCGTTAGCTTC
Lcn2 realtime for	CCAGTTCGCCATGGTATTTT
Lcn2 realtime reverse	CACACTCACCACCCATTCAG
Osmr realtime for	GTGAAGGACCCAAAGCATGT
Osmr realtime reverse	GCCTAATACCTGGTGCGTGT
S1pr3 realtime for	AAGCCTAGCGGGAGAGAAAC
S1pr3 realtime reverse	TCAGGGAACAATTGGGAGAG
Serpina3n realtime for	CCTGGAGGATGTCCTTTCAA
Serpina3n realtime reverse	TTATCAGGAAAGGCCGATTG
Steap4 realtime for	CCCGAATCGTGTCTTTCCTA
Steap4 realtime reverse	GGCCTGAGTAATGGTTGCAT
Timp1 realtime for	AGTGATTTCCCCGCCAACTC
Timp1 realtime reverse	GGGGCCATCATGGTATCTGC
Vim realtime for	AGACCAGAGATGGACAGGTGA
Vim realtime reverse	TTGCGCTCCTGAAAAACTGC
Hspb1 realtime for	GACATGAGCAGTCGGATTGA
Hspb1 realtime reverse	GGATGGGGTGTAGGGGTACT
H2-T23 realtime for	GGACCGCGAATGACATAGC
H2-T23 realtime reverse	GCACCTCAGGGTGACTTCAT
Serping1 realtime for	ACAGCCCCCTCTGAATTCTT
Serping1 realtime reverse	GGATGCTCTCCAAGTTGCTC
H2-D1 realtime for	TCCGAGATTGTAAAGCGTGAAGA
H2-D1 realtime reverse	ACAGGGCAGTGCAGGGATAG
Ggta1 realtime for	GTGAACAGCATGAGGGGTTT
Ggta1 realtime reverse	GTTTTGTTGCCTCTGGGTGT
Iigp1 realtime for	GGGGCAATAGCTCATTGGTA
Iigp1 realtime reverse	ACCTCGAAGACATCCCCTTT

Gbp2 realtime for	GGGGTCACTGTCTGACCACT
Gbp2 realtime reverse	GGGAAACCTGGGATGAGATT
Fbln5 realtime for	CTTCAGATGCAAGCAACAA
Fbln5 realtime reverse	AGGCAGTGTCAGAGGCCTTA
Ugt1a realtime for	CCTATGGGTCACTTGCCACT
Ugt1a realtime reverse	AAAACCATGTTGGGCATGAT
Fkbp5 realtime for	TATGCTTATGGCTCGGCTGG
Fkbp5 realtime reverse	CAGCCTTCCAGGTGGACTTT
Psmb8 realtime for	CAGTCCTGAAGAGGCCTACG
Psmb8 realtime reverse	CACTTTCACCCAACCGTCTT
Srgn realtime for	GCAAGGTTATCCTGCTCGGA
Srgn realtime reverse	TGGGAGGGCCGATGTTATTG
Amigo2 realtime for	GAGGCGACCATAATGTCGTT
Amigo2 realtime reverse	GCATCCAACAGTCCGATTCT
Clcf1 realtime for	CTTCAATCCTCCTCGACTGG
Clcf1 realtime reverse	TACGTCGGAGTTCAGCTGTG
Tgm1 realtime for	CTGTTGGTCCCGTCCCAAA
Tgm1 realtime reverse	GGACCTTCCATTGTGCCTGG
Ptx3 realtime for	AACAAGCTCTGTTGCCCATT
Ptx3 realtime reverse	TCCCAAATGGAACATTGGAT
S100a10 realtime for	CCTCTGGCTGTGGACAAAAT
S100a10 realtime reverse	CTGCTCACAAGAAGCAGTGG
Sphk1 realtime for	GATGCATGAGGTGGTGAATG
Sphk1 realtime reverse	TGCTCGTACCCAGCATAGTG
Cd109 realtime for	CACAGTCGGGAGCCCTAAAG
Cd109 realtime reverse	GCAGCGATTTCGATGTCCAC
Ptgs2 realtime for	GCTGTACAAGCAGTGGCAAA
Ptgs2 realtime reverse	CCCCAAAGATAGCATCTGGA
Emp1 realtime for	GAGACACTGGCCAGAAAAGC
Emp1 realtime reverse	TAAAAGGCAAGGGAATGCAC
Slc10a6 realtime for	GCTTCGGTGGTATGATGCTT
Slc10a6 realtime reverse	CCACAGGCTTTTCTGGTGAT
Tm4sf1 realtime for	GCCCAAGCATATTGTGGAGT
Tm4sf1 realtime reverse	AGGGTAGGATGTGGCACAAG
B3gnt5 realtime for	CGTGGGGCAATGAGAACTAT
B3gnt5 realtime reverse	CCCAGCTGAACTGAAGAAGG
Cd14 realtime for	GGACTGATCTCAGCCCTCTG
Cd14 realtime reverse	GCTTCAGCCCAGTGAAAGAC
sgNfe2l2-1 for	CACCGAAGACAAGAGCAACTCCAGA
sgNfe2l2-1 reverse	AAACTCTGGAGTTGCTCTTGTCTTC
sgNfe2l2-2 for	CACCGCTGGGCCGGCTGAATTGGG
sgNfe2l2-2 reverse	AAACCCCAATTCAGCCGGCCCAGC
mNfe2l2 DP for	ACCATGGCTTCTCCTTCCTT
mNfe2l2 DP reverse	CGACGAGTGTACCTGGGAGT
sgSirt1-1 for	CACCGAGAGACGGCTGGAACTGTCC

sgSirt1-1 reverse	AAACGGACAGTTCCAGCCGTCTCTC
sgSirt1-2 for	CACCGATACCTTGGAGCAGGTTGC
sgSirt1-2 reverse	AAACGCAACCTGCTCCAAGGTATC
sgSirt1-3 for	CACCGATTCATAGCTTTGTCAGATA
sgSirt1-3 reverse	AAACTATCTGACAAAGCTATGAATC
sgSirt1-4 for	CACCGCATGACACTGAAGGATCCTT
sgSirt1-4 reverse	AAACAAGGATCCTTCAGTGTCATGC
mSirt1 DP for	AGCTTTTTCTAGGGGCCATC
mSirt1 DP reverse	TGAGGCCTTCAGCTTTGTTT
sgScram for	CACCGCACTCACATCGCTACATCA
sgScram reverse	AAACTGATGTAGCGATGTGAGTGC
mIL-6 RT for	CCCCAATTTCCAATGCTCTCC
mIL-6 RT reverse	CGCACTAGGTTTGCCGAGTA
mTNF-α RT for	CCCCAGGGACCTCTCTCTAATC
mTNF-α RT reverse	GGTTTGCTACAACATGGGCTACA
mIL-1β RT for	TGCCACCTTTTGACAGTGATG
mIL-1β RT reverse	TGATGTGCTGCTGCGAGATT
mNOS2 RT for	CAGCTGGCCAATGAGGTACT
mNOS2 RT reverse	GCTCAAGTTCAGCTTGGTGG
mArg1 RT for	ACATTGGCTTGCGAGACGTA
mArg1 RT reverse	ATCACCTTGCCAATCCCCAG
mIL-5 RT for	AGGCTTCCTGTCCCTACTCA
mIL-5 RT reverse	CCCCCACGGACAGTTTGATT
mTGFβ2 RT for	AACACCCTCTGGCTCATTGG
mTGFβ2 RT reverse	CTCTGGCTTTGGGGTTTTGC
mCXCL5 RT for	CACTCGCAGTGGAAAGAACG
mCXCL5 RT reverse	CGTGGGTGGAGAGAATCAGC
mCXCL9 RT for	TAGAGCCCCTGCACACATTG
mCXCL9 RT reverse	CGAAAGCTACGTGGGAGGTT
mCCL17 RT for	CAATGTAGGCCGAGAGTGCT
mCCL17 RT reverse	CCTGGAACACTCCACTGAGG
mCCL20 RT for	GTGGGTTTCACAAGACAGATG
mCCL20 RT reverse	TTTTCACCCAGTTCTGCTTTG
mNrf2 RT for	TGAAGCTCAGCTCGCATTGA
mNrf2 RT reverse	TGCTCCAGCTCGACAATGTT
mNqo1 RT for	CATTGCAGTGGTTTGGGGTG
mNqo1 RT reverse	TCTGGAAAGGACCGTTGTCG
mHO-1 RT for	CCTCACAGATGGCGTCACTT
mHO-1 RT reverse	TGGGGGCCAGTATTGCATTT
mGclc RT for	GTGGACACCCGATGCAGTAT
mGclc RT reverse	GGTTGCACTTCCAAATGAGGC
mSrxn1 RT for	ACTATTCCTTTGGGGGCTGC
mSrxn1 RT reverse	GCTTGGCAGGAATGGTCTCT
mxCT RT for	TCAAAAGCTTGGCCATCTGC
mxCT RT reverse	TGCCTCACTGTATGACTTGCAT

mC3 for	CCAGCTCCCCATTAGCTCTG
mC3 reverse	GCACTTGCCTCTTTAGGAAGTC
mSirt1 RT for	GATACGGAGAGGCCCGAATG
mSirt1 RT reverse	AGTTCCCAATGCTGGTGGAG
mGAPDH RT for	CCTGGAGAAACCTGCCAAGTA
mGAPDH RT reverse	ACCAGGAAATGAGCTTGACA
GFAP promoter for	GCTAGCCCTGCAGGGAACATATCCTGGTGTGGAGTAG
GFAP promoter reverse	TTCGAAGGCGCGCCGCGAGCAGCGGAGGTGATGC
